# Safety and efficacy of endoscopic vs. microscopic approaches in pituitary adenoma surgery: A systematic review and meta-analysis

**DOI:** 10.1007/s10143-025-03600-3

**Published:** 2025-06-01

**Authors:** Nada Mostafa Al-dardery, Abdulrhman Khaity, Youssef Soliman, Mohamed Osama Mohamed Ali, Esraa Mohamed Zedan, Kamila Muyasarah, Mohamed Diaa Elfakhrany

**Affiliations:** 1 Faculty of Medicine, Fayoum University, Fayoum, Egypt; 2 Faculty of Medicine, Elrazi University, Khartoum, Sudan; 3 Faculty of Medicine, Assiut University, Assiut, Egypt; 4 Faculty of Medicine, Al-Azhar University, Cairo, Egypt; 5 Harvard Medical School, Harvard University, Boston, USA; 6 Neurosurgery Department, Faculty of Medicine, Fayoum University, Fayoum, Egypt

**Keywords:** Pituitary adenomas, Transsphenoidal surgery, Endoscopic, Microscopic

## Abstract

Pituitary adenomas (PAs) represent a prevalent category of intracranial tumors, frequently resulting in endocrine dysfunction and neurological impairments. Transsphenoidal surgery (TSS) serves as the primary treatment modality, with the endoscopic transsphenoidal approach (ETSA) and microscopic transsphenoidal approach (MTSA) representing the two principal techniques. This systematic review and meta-analysis aimed to assess and compare the outcomes of ETSA and MTSA in treating PAs. A literature search was performed across PubMed, Scopus, Web of Science, and Cochrane Library. A total of 31 studies comprising 38,301 patients were included. The primary outcomes assessed were gross total resection (GTR) and cerebrospinal fluid (CSF) leak rates. Secondary outcomes encompass endocrine and surgical complications and mortality rates. Statistical analyses utilized R software, employing random-effects models. The analysis indicated insignificant differences in GTR rates between ETSA and MTSA (RR: 1.05, 95% CI [0.97, 1.15]) or in CSF leak rates (RR: 1.03, 95% CI [0.82, 1.31]). Meta-regression analysis revealed that nonfunctional tumors correlated with increased GTR rates, regardless of the surgical technique employed. No notable differences were detected in endocrine or surgical complications between the two methods. Notably, meta-regression analysis indicated that nonfunctional tumors and higher Knosp grade tumors are more likely to be completely resected. Moreover, larger preoperative tumor volume was significantly correlated with an increased risk of postoperative hypopituitarism. Nevertheless, a narrative review of total mortality demonstrated a higher rate in the MTSA group compared to the ETSA group (198 vs. 149 deaths), even with a larger patient cohort in the ETSA group. No publication bias was observed for GTR or CSF leak outcomes. ETSA and MTSA exhibit similar efficacy and safety profiles in treating PAs, with no significant differences in resection rates or complications. The findings highlight the necessity of personalized surgical planning, considering tumor characteristics and institutional expertise. Future research should prioritize long-term outcomes, patient-reported metrics, and the incorporation of emerging technologies to enhance surgical strategies for PAs.

**Supplementary Information:**

The online version contains supplementary material available at 10.1007/s10143-025-03600-3.

## Introduction

Pituitary adenoma (PA) is an endocrine tumor that arises in the anterior or posterior pituitary gland, originating from residual craniopharyngioma epithelium. Epidemiological data suggest it is the third most prevalent brain tumor, following glioma and meningioma[38, 54] and accounts for 16.7% of intracranial tumors[42]. PA interferes with the secretion of growth hormone and thyrotropin, and its optic nerve compression may result in vision impairment. Surgical tissue damage may elicit stress responses, hindering recovery[5]. PA is categorized based on hormonal activity into non-secreting, prolactin-secreting (PRL), and producing adrenocorticotropic hormone (ACTH), growth hormone (GH), or thyroid-stimulating hormone (TSH). Since medication often leads to undesirable side effects, transsphenoidal surgery (TSS) remains a well-established treatment option, with continuous advancements over the past decade[20, 37]. Nonfunctioning pituitary adenomas (NFPAs) are the most prevalent type, following prolactinomas. NFPAs constitute one-third of all PAs and are primarily characterized by tumor mass effects, lacking hormonal hypersecretion[7].

Surgery serves as the principal treatment for pituitary adenomas, which can be executed via craniotomy or transsphenoidal approach[41]. A craniotomy is indicated for larger tumors or those that extend superiorly and laterally beyond the sella. The TSS is preferable for tumors localized to the sella or those extending into the sphenoid sinus, especially in patients with well-pneumatized sphenoid sinuses[18]. The TSS for sellar tumor resection was initially introduced by Schoffler in 1907 and subsequently refined by Cushing. In the 1960 s, Jules Hardy's implementation of the operating microscope markedly improved surgical precision. Since the early twenty-first century, advancements in endoscopic techniques have enhanced visualization and outcomes, resulting in widespread adoption[3]. Despite advancements in pituitary adenoma surgery, the optimal approach between the endoscopic transsphenoidal approach (ETSA) and the microscopic transsphenoidal approach (MTSA) remains unclear. ETSA enhances visualization[4, 13]; however, it introduces a learning curve and potential risks, including prolonged operative time and cerebrospinal fluid (CSF) leaks[40]. Conversely, MTSA may result in extended hospitalizations and increased nasal disruption[30, 34]^,^[24]. The lack of standardized protocols and objective comparative research underscores the necessity for high-quality studies to identify the most effective surgical technique for managing PAs.

This systematic review and meta-analysis aimed to compare the safety and efficacy of ETSA versus MTSA for treating PA. The analysis will concentrate on essential outcomes, such as the gross total resection (GTR) rate, endocrinological results, surgical complications, and mortality rates, to offer evidence-based insights into the most effective surgical approach.

## Materials and methods

This meta-analysis adhered to the PRISMA guidelines for systematic reviews and meta-analyses[45]. All procedures adhered to the guidelines specified in the Cochrane Handbook for Systematic Reviews of Interventions, version 6.3 [14]. The study protocol received registration in PROSPERO with the registration ID CRD420250653732.

### Literature search strategy

A comprehensive search was conducted across four major electronic databases—PubMed, Scopus, Web of Science, and Cochrane Library—from their inception until January 2025. The search strategy included keywords such as "transsphenoidal", "surgery", "endoscopic", "microscopic", and "pituitary", and their synonyms, facilitating a comprehensive and targeted literature retrieval.

### Eligibility criteria

Eligible studies comprised full-text comparative analyses of ETSA and MTSA in patients diagnosed with pituitary adenomas, including functioning and nonfunctioning types. Additionally, studies needed to report at least one predefined primary or secondary outcome to ensure alignment with the research objectives. Only articles published in English were included to maintain consistency in data interpretation. Non-comparative studies, including reviews, single-arm studies, case reports, conference abstracts, and case series, were excluded from the analysis. Studies that did not specifically address pituitary adenomas, failed to assess relevant surgical or endocrinological outcomes, or were published in languages other than English were excluded from the analysis.

### Outcome interests

The primary outcomes consist of the GTR rate and the occurrence of postoperative CSF leaks. Secondary outcomes include endocrine complications such as postoperative hypopituitarism, adrenal insufficiency, hyponatremia, and diabetes insipidus (DI); surgical complications including visual deterioration, meningitis, carotid artery injury, intracranial hemorrhage (ICH), and Epistaxis; as well as mortality rates.

### Study selection

All duplicate records were eliminated utilizing EndNote software. Eligibility was assessed through a two-step screening process involving an initial review of titles and abstracts, preceded by a full-text evaluation. Two reviewers independently evaluated each study, while a third addressed any discrepancies. We also manually reviewed the reference lists from the included studies to identify and include any pertinent articles that met our eligibility criteria.

### Data extraction

Excel spreadsheets were employed to extract essential data, encompassing systematically 1) baseline characteristics of study populations, 2) summaries of included studies, 3) quality assessment domains, and 4) study outcomes such as; GTR, postoperative CSF leak, endocrine complications (postoperative hypopituitarism, adrenal insufficiency, hyponatremia, and DI), surgical complications (visual worsening, meningitis, injury to the carotid artery, ICH, and Epistaxis) and mortality rates.

### Risk of bias assessment

The Newcastle–Ottawa Scale (NOS) [52] was used to assess the quality of double-arm observational studies. The single randomized controlled trial (RCT) included was assessed using Version 2 of the Cochrane Risk-of-Bias Tool (ROB 2) [53], following the guidelines specified in the Cochrane Handbook for Systematic Reviews of Interventions (version 6.3). The tools facilitated a thorough and uniform evaluation of methodological quality for all studies included.

### Statistics analysis

Statistical analyses were performed using R software, employing the inverse variance method. Continuous variables were aggregated as mean differences (MDs) accompanied by 95% confidence intervals (CIs). In contrast, dichotomous variables were assessed using relative risk (RR) or odds ratios (ORs) within a random-effects model. Heterogeneity was evaluated using I^2^ statistics and chi-squared (χ^2^) tests, with a χ^2^ *p*-value < 0.1 indicating significant heterogeneity. A *p*-value less than 0.05 was deemed statistically significant. A meta-regression analysis assessed the impact of key demographic and clinical factors, such as age, sex, and adenoma type (functioning vs. nonfunctioning), on surgical and postoperative outcomes. In addition, we performed a sensitivity analysis to evaluate the robustness of our results. This entailed reconsidering the data under varying assumptions to assess the potential influence of heterogeneity and methodological decisions on the overall outcomes. Furthermore, we visually inspected funnel plots for asymmetry to determine the probability of bias from missing results in the synthesis, which may indicate publication bias or selective reporting. Additionally, Egger's test was conducted to evaluate small-study effects, which may indicate bias if significant.

## Results

### Literature search

**Fig. 1:**
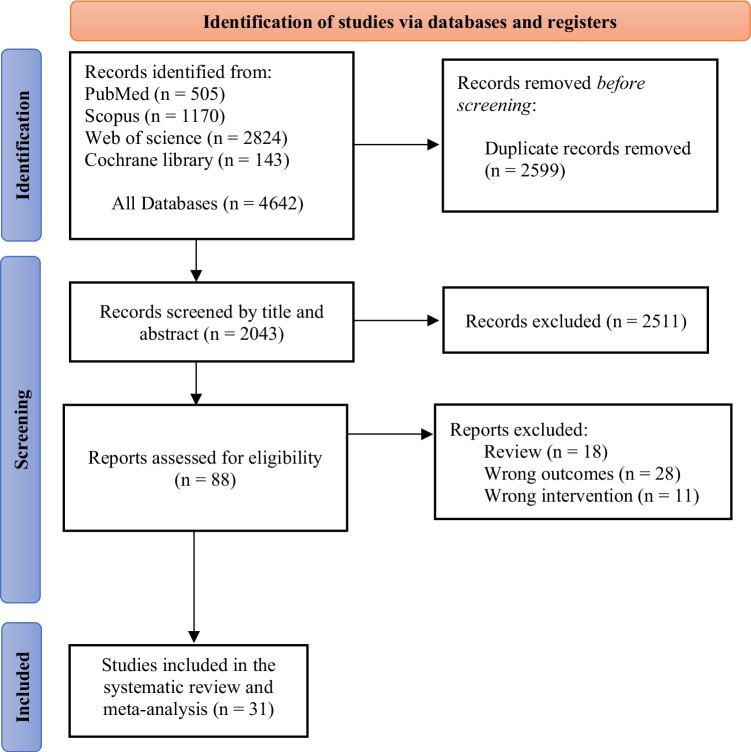
The PRISMA flow diagram

Our search strategy yielded a total of 4642 records. Before the screening, 2,599 duplicate records were eliminated. A total of 2,043 studies were screened by title and abstract, excluding 2,511 records. After the initial screening, 88 full-text reports were evaluated for eligibility, resulting in 57 further exclusions. The exclusions included 18 reviews, 28 instances of incorrect outcomes, and 11 cases of inappropriate interventions. Ultimately, 31 studies met our criteria and were included in our systematic review and meta-analysis. Figure 1 shows the PRISMA flow diagram of the present study.

### Characteristics and quality of the included studies

Key findings from 30 cohort studies and one RCT are detailed in Table 1. This table summarizes studies comparing ETSA and MTSA for PA surgery across 15 countries regarding surgical outcomes for diverse illnesses, with a primary emphasis on tumor removal. Follow-up intervals are spanning from 3 to 120 months. Research encompasses sample sizes ranging from 20 to 30,488 patients, aggregating to 38,301 individuals. Baseline characteristics of the included population are shown in *Online Resource* 2.

**Table 1:** Summary of the included studies

Study	Country	Study design	N	Follow-up months	Key findings
Prajapati et al., 2018[47]	India	Cohort Prospective	30	NR	Endoscopy led to reduced blood loss, decreased operative duration, and a lower incidence of complications
Zaidi et al., 2016[58]	USA	Cohort Prospective	135	6 months	The ETSA may offer specific benefits in expediting the learning curve associated with pituitary surgery
Trimpou et al., 2022[55]	Sweden	Cohort Retrospective	40	89 months	ETSA and MTSA for Cushing's disease exhibit comparable rates of remission, recurrence, and complications; however, the endoscopic method is linked to a reduced hospital stay owing to its minimally invasive nature
Phogat et al., 2020[46]	India	Cohort Prospective and Retrospective	198	6 months	Endoscopy had higher GTR rates and fewer complications, but longer operative time
Song et al., 2022[51]	China	Cohort Retrospective	514	43.5 months	ETSA presents an increased risk of CSF leakage; however, it enhances the resection of nonfunctional adenomas exhibiting suprasellar extension or compression of the optic chiasm
Goshtasbi et al., 2021[23]	USA	Cohort Retrospective	30488	NR	There has been an increase in the use of ETSA over time, resulting in a shorter stay while maintaining comparable GTR rates
Pablo et al., 2019[44]	Argentina	Cohort Retrospective	399	12 months	Endoscopy demonstrated superior outcomes for invasive adenomas; however, there was no notable difference in complications
Eseonu et al., 2017[17]	USA	Cohort Retrospective	384	120 months	Reduced operative time and hospital stay in ETSA, while maintaining comparable EOR
Findlay et al., 2023[19]	USA	Cohort Retrospective	600	6 months	In MTSA, there is a higher GTR; however, this is associated with increased ICU stays and complications
Agam et al., 2018[1]	USA	Cohort Retrospective	1153	NR	No notable differences in complications were observed; however, prior surgery and tumor invasion emerged as risk factors
Akbari et al., 2018[2]	Iran	Cohort Retrospective	35	6 months	The higher GTR in ETSA is 81.2%, compared to 15.8%, with comparable complications
Casler et al., 2005[8]	USA	Cohort Retrospective	30	NR	The endoscopic approach reduced hospital stay, decreased blood loss, and expedited recovery
Cheng et al., 2011[9]	China	Cohort Retrospective	127	24 months	Improved disease management in ETSA, particularly concerning macroadenomas
Gao et al., 2016[21]	China	Cohort Retrospective	105	7 months	ETSA demonstrates a higher GTR and reduced complications but is associated with an extended operative duration
Higgins et al., 2012[27]	USA	Cohort Retrospective	48	20 months	ETSA demonstrated reduced operative durations, decreased blood loss, and abbreviated hospital stays
Hong et al., 2015[28]	USA	Cohort Retrospective	55	3 months	Comparable olfactory results, yet improved early sinonasal quality of life in MTSA
Huang et al., 2023[29]	China	Case–control study	127	NR	ETSA results in reduced complications and improved symptom relief; however, it is associated with a longer operative duration
Little et al., 2019[35]	US	Cohort Prospective	260	6 months	Comparable GTR rates, but ETSA has less hormone insufficiency
Qiao et al., 2021[48]	China	Cohort Retrospective	1118	6 months	Endoscopy correlated with increased rates of endocrine remission and a reduction in CSF leaks
Razak et al., 2012[49]	UK	Cohort Retrospective	80	6 months	MTSA is considered the gold standard; however, preliminary results suggest that ETSA may provide advantageous outcomes in tumor resection and hormonal regulation
Shimony et al., 2021[50]	Israel	Cohort Retrospective	684	24 months	ETSA for NFPMA demonstrates a reduced probability of reoperation relative to the microscopic technique while maintaining comparable rates of EOR and complications
Gompel et al., 2021[56]	US	Cohort Retrospective	534	27 months	ETSA involves a higher volumetric resection, whereas MTSA is characterized by shorter operative times and reduced costs
Karppinen et al., 2015[33]	Finland	Cohort Retrospective	185	12 months	Higher GTR in ETSA, but longer operative time​
Messerer et al., 2011[39]	France	Cohort Retrospective	164	12 months	Endoscopy enhanced GTR and endocrine outcomes, particularly regarding lateral extension
Dallapiazza et al., 2014[16]	US	Cohort Retrospective	99	12 months	Resection rates were comparable; however, intraoperative CSF leaks were more prevalent in ETSA
Choe et al., 2008[12]	Korea	Cohort Retrospective	23	12 months	ETSA demonstrated higher imaging and hormonal remission rates, while the incidence of CSF leakage remained comparable
D'Haens et al., 2009[15]	Belgium	Cohort Retrospective	120	18 months	Increased remission rates in ETSA for macroadenomas are associated with a higher incidence of CSF leaks
O'Malley et al., 2008[43]	US	Cohort Retrospective	50	8 months	Endoscopic endonasal resection demonstrates similar complication rates and symptom resolution outcomes compared to conventional surgical methods
Jain,2007[31]	India	Randomized Clinical Trial	20	6.95 months	ETSA demonstrated reduced blood loss and fewer complications while maintaining comparable resection rates
Kahilogullari et al., 2013[32]	Turkey	Cohort Prospective	50	6 months	ETSA demonstrated superior preservation of olfactory function
Halvorsen et al., 2013[26]	Norway	Cohort Prospective and Retrospective	446	6 months	There is no notable difference in complication rates among the various approaches

Regarding the risk of bias evaluation in 30 cohort studies utilizing the NOS, twenty-six studies received a good rating, reflecting strong methodology. In contrast, three studies were rated fair due to moderate bias, and one was rated poor due to significant bias. The included RCT was evaluated as having some concerns about bias. Details of the assessment for each single study are presented in *Online Resources* 3, 4.

### Meta-analysis

#### Primary outcomes

**Fig. 2:**
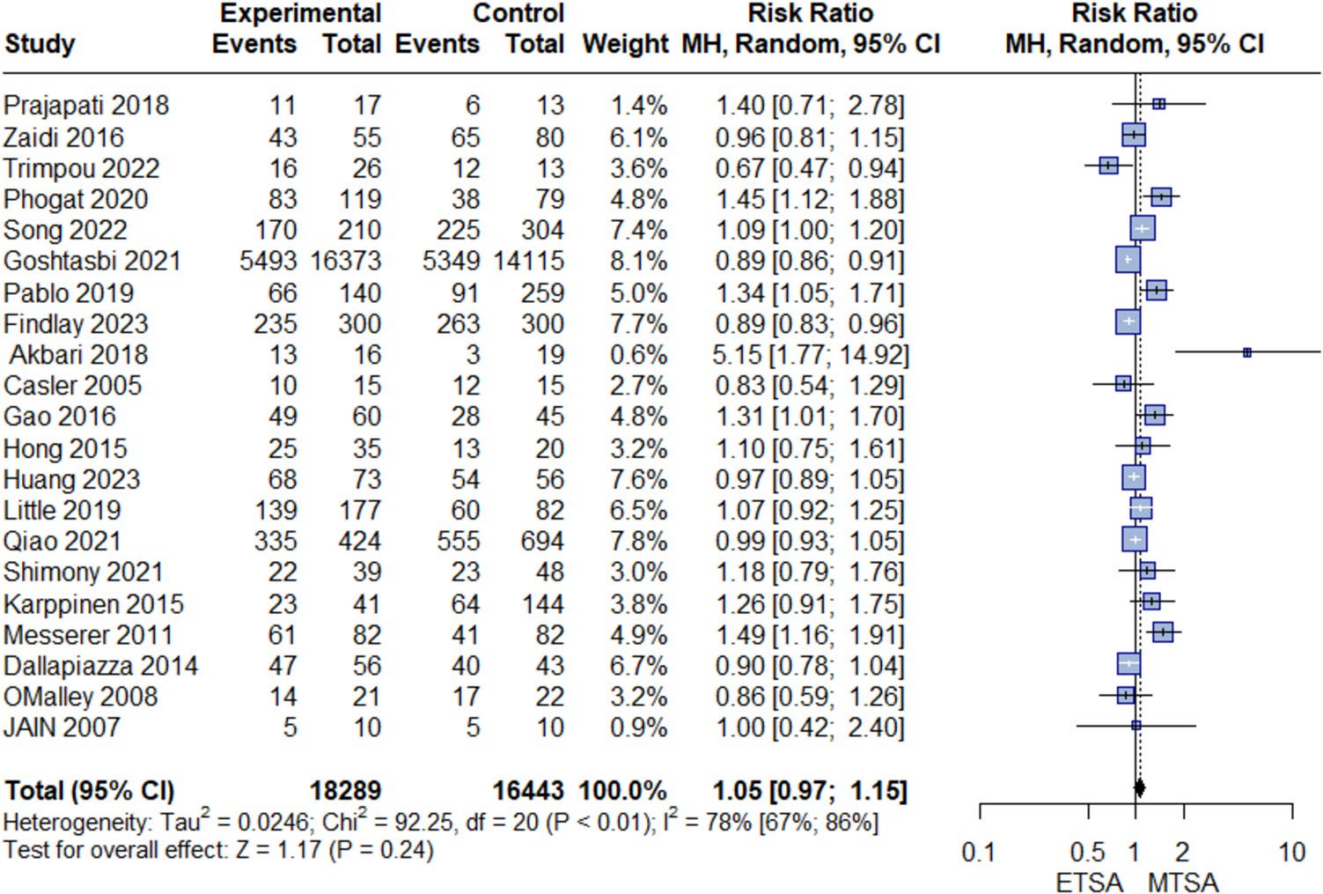
Forest plot of GTR

**Fig. 3:**
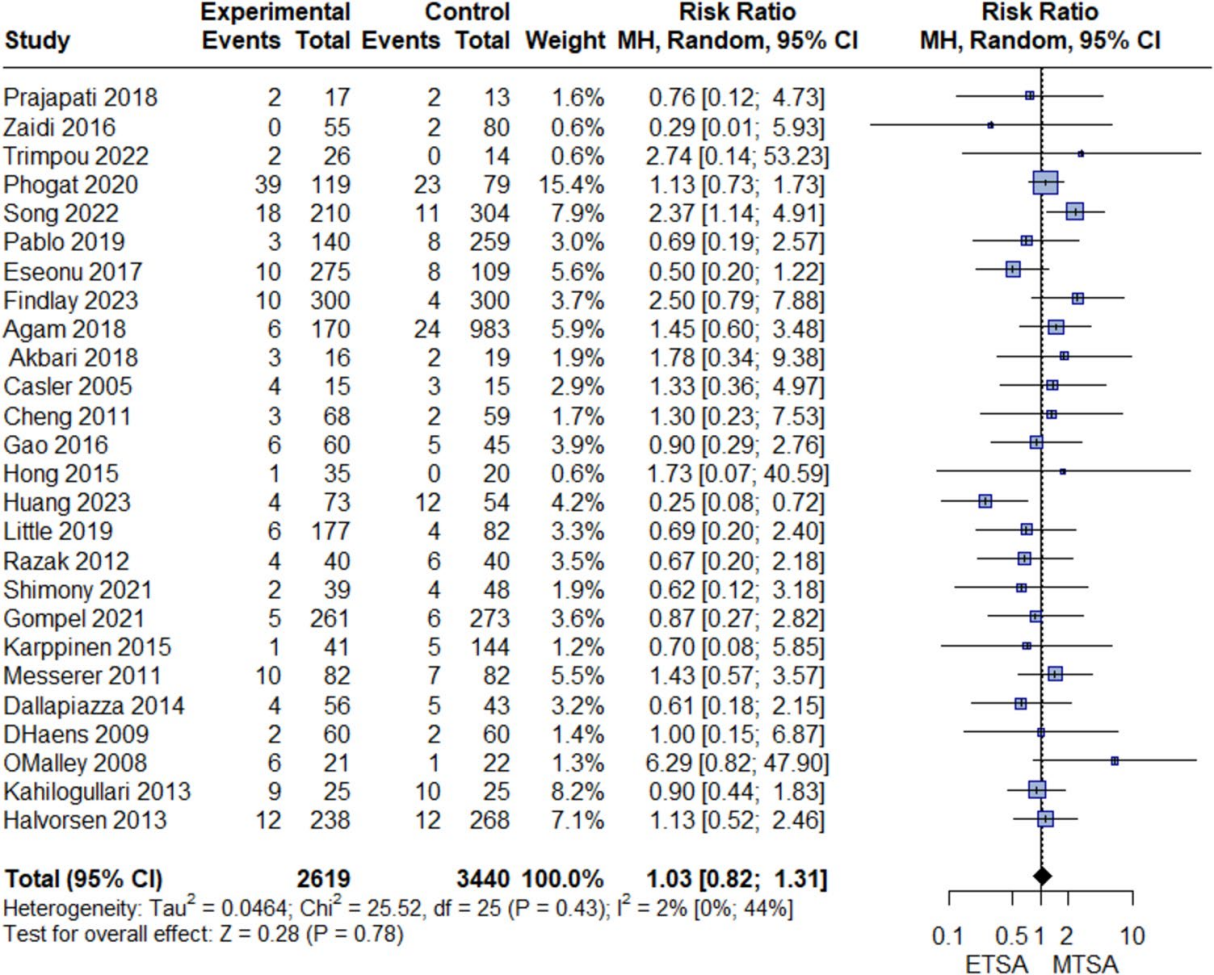
Forest plot of postoperative CSF leak

No difference was between ETSA and MTSA in GTR (RR: 1.05, 95% CI [0.97, 1.15], I^2^ = 78%), Fig. 2. Sensitivity analysis did not resolve the heterogeneity, *Online resource 1, *Fig. 1. Similarly, we found no difference in postoperative CSF leak between the two groups (RR: 1.03, 95% CI [0.82, 1.31], I^2^ = 2%), Fig. 3*.* We assessed publication bias for both GTR and CSF leaks. By inspecting the funnel plot and Egger's test, we found no significant publication bias (*P*-value > 0.05), *Online resource 1, *Fig. 2*.*

#### Endocrine complications

**Fig. 4:**
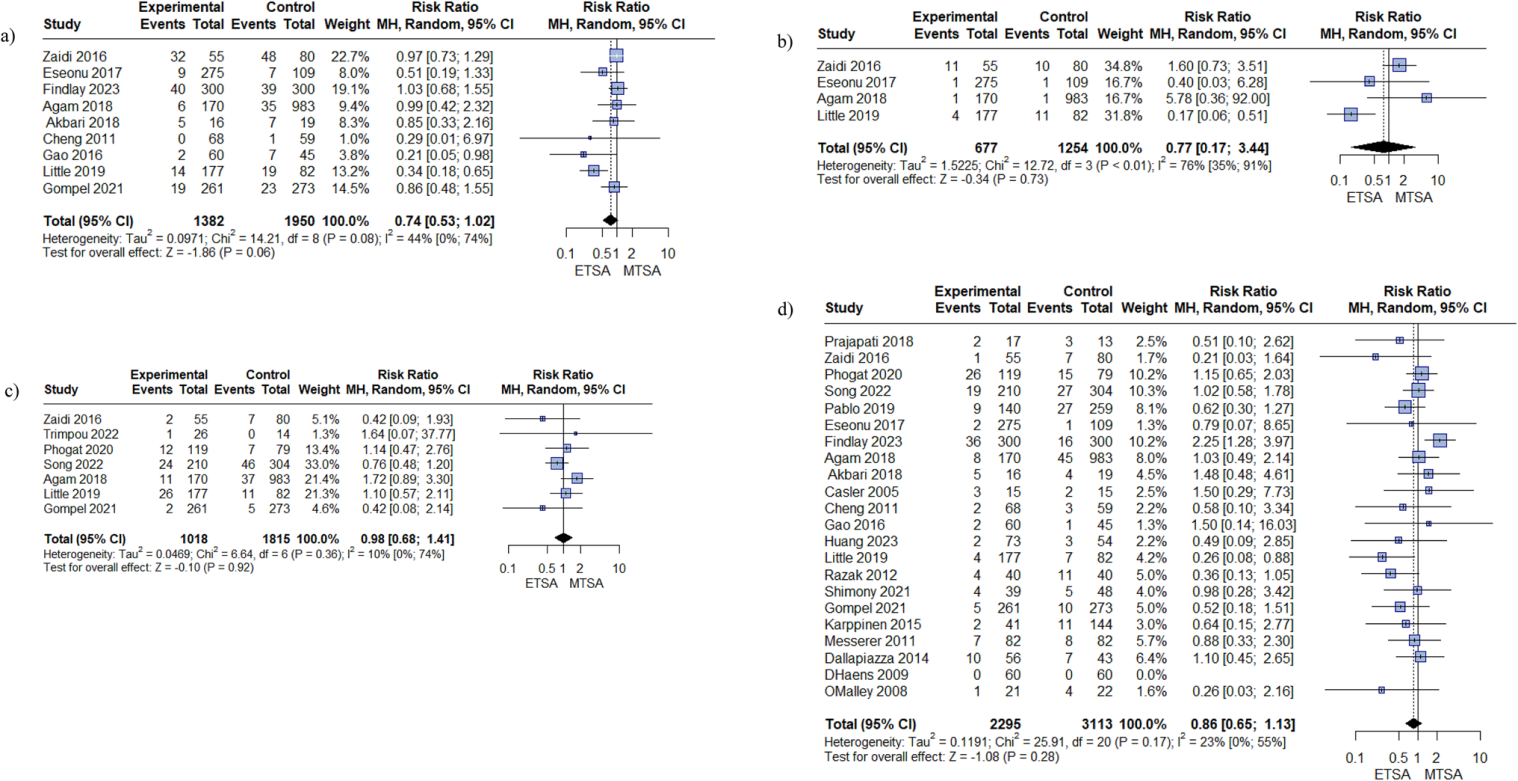
Forest plots of postoperative endocrine complications

There was no difference between the two arms in postoperative hypopituitarism (RR: 0.74, 95% CI [0.53, 1.02], I^2^ = 44%) Fig. 4, a, adrenal insufficiency (RR: 0.77, 95% CI [0.17, 3.44], I^2^ = 76%) Fig. 4,b, hyponatremia (RR: 0.98, 95% CI [0.68, 1.41], I^2^ = 10%) Fig. 4,c, DI (RR: 0.86, 95% CI [0.65, 1.13], I^2^ = 23%) Fig. 4,d.

#### Surgical complications

Similar to endocrine complications, there was no difference between the two interventions in surgical complications like visual worsening (RR: 1.41, 95% CI [0.97, 1.53], I^2^ = 0%), meningitis (RR: 0.90, 95% CI [0.69, 1.17], I^2^ = 6%), carotid injury (RR: 0.82, 95% CI [0.07, 17.90], I^2^ = 0%), ICH (RR: 0.70, 95% CI [0.28, 1.74], I^2^ = 0%), and epistaxis (RR: 1.30, 95% CI [0.66, 2.57], I^2^ = 0%) Online resource 1, Fig. 3–7.

#### Meta-regression analysis

**Fig. 5:**
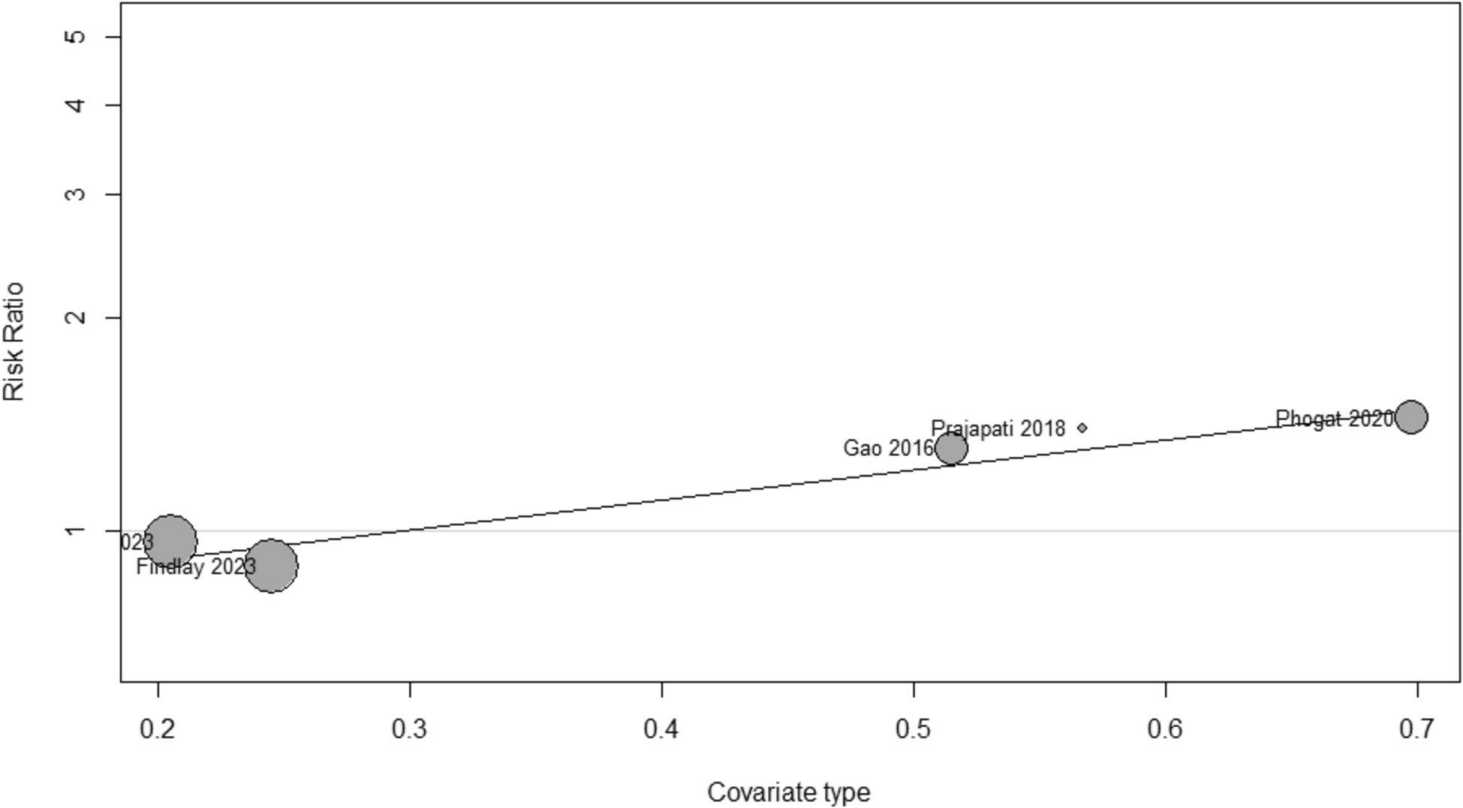
Meta-regression for GTR according to type of pituitary adenoma

A meta-regression analysis assessed the influence of age, sex, tumor type (functional or nonfunctional), preoperative tumor volume, and Knosp grade on surgical outcomes. The study indicated that the proportion of patients with nonfunctional tumors significantly predicted the effect size. A higher percentage of nonfunctional tumors correlated with an increased likelihood of achieving GTR (Beta = 0.98, SE = 0.29, *P* = 0.006), indicating that nonfunctional tumors may be more appropriate for complete resection (Fig. 5). A higher Knosp grade, which generally shows more invasive tumors, was positively correlated with GTR (Beta = 1.005, SE = 0.46, *P* = 0.02). This association may reflect the implementation of aggressive surgical strategies for more invasive lesions. Larger preoperative tumor volume was significantly correlated with an increased risk of postoperative hypopituitarism (Beta = 0.14, SE = 0.05, *P* = 0.003). Nonetheless, no significant associations were identified between the examined predictors and the risks of DI, visual deterioration, meningitis, or CSF leak (all *P*-values > 0.05), suggesting insufficient evidence to connect these variables to the specified postoperative outcomes Online resource 5.

### Systematic review of total mortality

The bar chart Online resource 1, Fig. 8, illustrates the comparison of patient numbers and overall mortality rates between ETSA and MTSA. The ETSA group comprised 18,130 patients, whereas the MTSA group comprised 16,678 patients. Although the ETSA group had a marginally greater number of patients, the MTSA group experienced a higher total mortality rate, with 198 deaths compared to 149 in the ETSA group. This indicates that the microscopic approach may be linked to a higher mortality rate than the endoscopic approach. The combined total for both groups was 34,808 patients and 347 deaths.

## Discussion

PAs, among the most prevalent brain tumors, often result in endocrine dysfunction and neurological impairments[10, 22, 36]. The standard treatment is TSS, which can be performed using either an ETSA or MTSA[10, 22, 36]. Despite their widespread use, there is still discussion over which method is more effective for complete tumor removal, postoperative complications, and long-term patient outcomes[3, 11, 25, 57]. This meta-analysis aimed to evaluate and compare the safety and efficacy of the ETSA versus MTSA in treating PAs.

Our meta-analysis incorporated 31 studies (30 cohort studies, 1 RCT) involving 38,301 patients. The findings of this study revealed no statistically significant difference between ETSA and MTSA in achieving GTR. However, meta-regression indicated that a higher percentage of patients with nonfunctional tumors was associated with a higher likelihood of GTR, irrespective of the surgical approach. This suggests that tumor characteristics play a significant role in resection success. In addition, a higher Knosp grade was positively correlated with GTR, which may reflect the implementation of aggressive surgical strategies for more invasive lesions.

Furthermore, the two surgical techniques did not differ significantly in terms of postoperative complications such as CSF leaks, endocrine issues (including hypopituitarism, adrenal insufficiency, hyponatremia, and DI), or other complications like visual deterioration, meningitis, carotid injury, ICH, and Epistaxis. However, meta-regression revealed that larger preoperative tumor volume was significantly correlated with an increased risk of postoperative hypopituitarism. Interestingly, the narrative review of mortality rate showed a higher incidence in the MTSA group than in the ETSA group, despite a marginally larger patient count in the ETSA group. Lastly, the absence of significant publication bias for the outcomes of GTR and CSF leaks reinforces the reliability of our meta-analysis findings.

In former studies, Gao et al. (2014)[22] reported superior GTR rates with ETSA, particularly for invasive adenomas, while Chen et al. (2020)[10] and Li et al. (2017)[36] found no significant difference in resection efficacy. Our findings align more closely with the latter, attributing discrepancies to heterogeneity in tumor subtypes and study designs. Meta-regression in our study revealed that nonfunctional adenomas, often larger and less hormonally active, were associated with higher GTR rates regardless of surgical approach, emphasizing the role of intrinsic tumor characteristics in resection success. This finding is supported by Yu et al. (2018)[57], who observed improved outcomes with ETSA in nonfunctioning tumors, and contrasts with Phogat et al. (2021)[46], whose single-center experience demonstrated significantly higher GTR rates with endoscopy, suggesting that enhanced visualization may favor complete tumor removal under specific conditions.

Regarding complications, our analysis found no significant differences in postoperative complications, including CSF leaks, endocrine disturbances (e.g., hypopituitarism, DI), or adverse events such as visual deterioration and meningitis. These results are consistent with Chen et al. (2020)[10] and Li et al. (2017)[36], as well as broader literature reviews by Guo et al. (2021)[25] and Chen et al. (2022)[10]. However, Gao et al. (2014)[22] have reported lower rates of DI, hypothyroidism, and septal perforation with ETSA, suggesting potential safety advantages. Additionally, Gao et al. (2014)[22] highlighted shorter hospital stays and reduced meningitis rates with ETSA. However, our analysis did not replicate these findings, possibly due to regional variations in antibiotic use or reporting practices.

The subgroup analyses in the literature further complicate the picture. Guo et al. (2021)[25] reported improved GTR and visual outcomes with ETSA in hormone-secreting tumors, while Broersen et al. (2018)[6] found superior outcomes for macroadenomas in Cushing's disease. These variations underscore the importance of individualized surgical planning, considering tumor size, type, and institutional expertise.

An intriguing aspect of our review was the higher mortality rate, which, narratively reviewed, was observed in the microscopic group compared to the endoscopic cohort. While this mortality difference was not consistently reported across all previous studies, it hints at potential survival benefits with the endoscopic approach, possibly due to reduced intraoperative trauma and improved preservation of neurovascular structures.

Although both ETSA and MTSA yield similar overall resection rates and safety profiles, subtle differences related to tumor characteristics and specific postoperative outcomes suggest that personalized treatment strategies are essential. These nuances highlight the potential benefits of the endoscopic approach in select clinical scenarios, reinforcing the need for tailored surgical decision-making in managing PAs.

Our findings reveal no significant difference between ETSA and MTSA regarding key surgical and endocrine outcomes, indicating that neither approach can be universally preferred. Consequently, we do not endorse a universal surgical approach. We propose a customized, patient-centered strategy considering various clinical and contextual factors. Characteristics specific to tumors, including size, functionality, and invasiveness, should inform surgical planning, in conjunction with the surgeon's expertise and familiarity with the respective technique. Institutional resources, such as available equipment and support systems, are essential in identifying the most suitable surgical method. This study underscores the importance of individualized decision-making, advocating for adaptable surgical strategies to enhance outcomes tailored to specific patient and center contexts.

Our study provides the most comprehensive and current analysis compared to earlier systematic reviews and meta-analyses by Gao et al. (2014)[22], Li et al. (2017)[36], and Chen et al. (2022)[10], featuring a substantially larger pooled sample size of 38,301 patients, which surpasses that of previous studies. All reviews consistently indicated no significant difference between ETSA and MTSA regarding key outcomes such as GTR, CSF leak, and meningitis. Our analysis further examined a wider array of postoperative complications, including adrenal insufficiency, hyponatremia, carotid injury, and intracerebral hemorrhage, all of which also demonstrated no significant differences between the surgical techniques. The incorporation of meta-regression analysis represents a considerable strength of our study, as it was absent in prior reviews. This identified a significant association between nonfunctional pituitary adenomas and higher Knosp grades with increased GTR rates. At the same time, larger preoperative tumor volumes correlated with a heightened risk of postoperative hypopituitarism. The findings provide important insights into patient- and tumor-specific factors affecting surgical outcomes, enhancing personalized planning in managing pituitary adenomas Table 2.

**Table 2:** Comparative table with the previously published systematic reviews

Study ID	Gao 2014[22]	Li 2017[36]	Chen 2022[10]	Our study
Number of Participants	1014	2272	5591	38301
Outcomes of Meta-Analysis				
GTR	Favor ETSA	Favor ETSA	No difference between ETSA and MTSA	No difference between ETSA and MTSA
Postoperative CSF leak	No difference between ETSA and MTSA	No difference between ETSA and MTSA	No difference between ETSA and MTSA	No difference between ETSA and MTSA
Postoperative hypopituitarism	No difference between ETSA and MTSA	No difference between ETSA and MTSA	NR	No difference between ETSA and MTSA
Postoperative adrenal insufficiency	NR	NR	NR	No difference between ETSA and MTSA
Postoperative hyponatremia	NR	NR	No difference between ETSA and MTSA	No difference between ETSA and MTSA
Postoperative DI	NR	No difference between ETSA and MTSA	Favor ETSA	No difference between ETSA and MTSA
Postoperative visual worsening	NR	NR	No difference between ETSA and MTSA	No difference between ETSA and MTSA
Postoperative meningitis	No difference between ETSA and MTSA	No difference between ETSA and MTSA	No difference between ETSA and MTSA	No difference between ETSA and MTSA
Postoperative carotid injury	NR	NR	NR	No difference between ETSA and MTSA
Postoperative ICH	NR	NR	NR	No difference between ETSA and MTSA
Postoperative Epistaxis	No difference between ETSA and MTSA	No difference between ETSA and MTSA	No difference between ETSA and MTSA	No difference between ETSA and MTSA
Outcomes of Meta-Regression (Age, Sex, Type of Pituitary adenoma, whether functioning or not functioning, preoperative tumor volume, and Knosp grade)				
GTR	NR	NR	NR	Nonfunctional tumors and higher Knosp grade are correlated with increased GTR rates
Postoperative CSF leak	NR	NR	NR	No significant correlation
Postoperative DI	NR	NR	NR	No significant correlation
Postoperative visual worsening	NR	NR	NR	No significant correlation
Postoperative meningitis	NR	NR	NR	No significant correlation
postoperative hypopituitarism	NR	NR	NR	Larger preoperative tumor volume correlated with an increased risk of postoperative hypopituitarism

## Future perspectives

The findings of our meta-analysis provide valuable insights into the comparative efficacy and safety of ETSA and MTSA for PAs. However, several key areas require further exploration to optimize surgical outcomes and guide clinical decision-making. Future research should prioritize the following directions: **1) Incorporating Patient-Centric Metrics:** Beyond clinical endpoints like resection rates, future work should integrate patient-reported outcomes (PROs) assessing nasal function, recovery duration, and hormonal restoration. Pairing PROs with biomarkers (e.g., post-op hormone assays) could optimize individualized care.; **2) Standardization of Complication Reporting:** Differences in reported complications, such as CSF leaks or meningitis, highlight the need for uniform definitions and reporting standards. Establishing collaborative, multicenter registries could reduce biases and improve the generalizability of future findings.; **3) Global and Socioeconomic Considerations:** Given the disparities in healthcare resources and surgical expertise worldwide, future studies should examine how institutional factors influence outcomes. Cost-effectiveness analyses comparing ETSA and MTSA are fundamental in low-resource settings, where access to advanced endoscopic equipment may be limited. Identifying cost-efficient and effective strategies is critical for global implementation.; and **4) Technological Advancements:** Emerging tools like 3D endoscopy, intraoperative MRI, and robotic-assisted systems could further optimize, etc. Comparative studies assessing these innovations against conventional techniques are needed to define their role in minimizing complications (e.g., CSF leaks) and improving resection precision.

Addressing these research priorities through rigorously designed randomized trials and collaborative multicenter studies will refine surgical decision-making and improve patient outcomes in treating pituitary adenomas. As surgical technology advances, integrating novel techniques, personalized treatment strategies, and standardized outcome measures will be essential for shaping the future of pituitary surgery.

## Limitations and strengths of the study

This meta-analysis has several limitations that warrant consideration. The included studies exhibited methodological heterogeneity, with variability in tumor characteristics, surgical techniques, and geographic settings, potentially confounding direct comparisons. Moreover, the absence of detailed stratification for specific subgroups, such as giant or functionally active adenomas, limits our ability to assess how tumor size and behavior affect surgical success. The predominance of observational cohort studies (30 out of 31) over RCTs further constrains causal interpretations, and the limited reporting on long-term outcomes (e.g., recurrence and quality-of-life) restricts our understanding of sustained efficacy. In addition, a significant limitation of this study is the representation of total mortality as a qualitative analysis instead of a quantitative meta-analysis. This resulted from inadequate and inconsistent mortality data reporting in the analyzed studies. Consequently, we could not conduct a comprehensive statistical synthesis or account for potential confounding variables.

Conversely, our study boasts several strengths. With a robust sample size exceeding 38,000 patients across 15 countries and including 31 studies, our analysis benefits from enhanced statistical power and a diverse patient population. Rigorous sensitivity analyses and meta-regression techniques have bolstered the reliability of our findings, particularly in clarifying the role of nonfunctional tumors in GTR outcomes. Importantly, no significant publication bias was detected, reinforcing the results'credibility. These strengths collectively provide valuable insights into the safety and efficacy of ETSA and MTSA, offering a solid foundation for future prospective studies and tailored surgical strategies.

## Conclusions

In conclusion, our meta-analysis of 31 studies encompassing over 38,000 patients revealed no significant differences between ETSA and MTSA in achieving gross total resection, CSF leak rates, or postoperative endocrine and surgical complications. Notably, meta-regression analysis indicated that nonfunctional tumors and higher Knosp grade tumors are more likely to be completely resected. Moreover, larger preoperative tumor volume was significantly correlated with an increased risk of postoperative hypopituitarism. Although the study's robust sample size and global representation enhance its credibility, the methodological heterogeneity and predominance of observational studies warrant cautious interpretation. Future prospective trials that stratify outcomes by tumor characteristics are essential to refine surgical strategies and confirm these results.

## Supplementary Information

Below is the link to the electronic supplementary material.

Supplementary file1 (PDF 271 KB)

Supplementary file2 (PDF 262 KB)

Supplementary file3 (PDF 246 KB)

Supplementary file4 (PDF 145 KB)

Supplementary file5 (PDF 85 KB)

## Data Availability

No datasets were generated or analysed during the current study.
