# Supplementary material for: Safety and efficacy of endoscopic vs. microscopic approaches in pituitary adenoma surgery: A systematic review and meta-analysis
Source: Neurosurg Rev. 2025 Jun 1;48(1):471. doi: 10.1007/s10143-025-03600-3 (PMC12126332; doi:10.1007/s10143-025-03600-3)
Supplement: Supplementary file 1 — Supplementary file1 (PDF 271 KB) [file 10143_2025_3600_MOESM1_ESM.pdf]

Supplementary Figures.

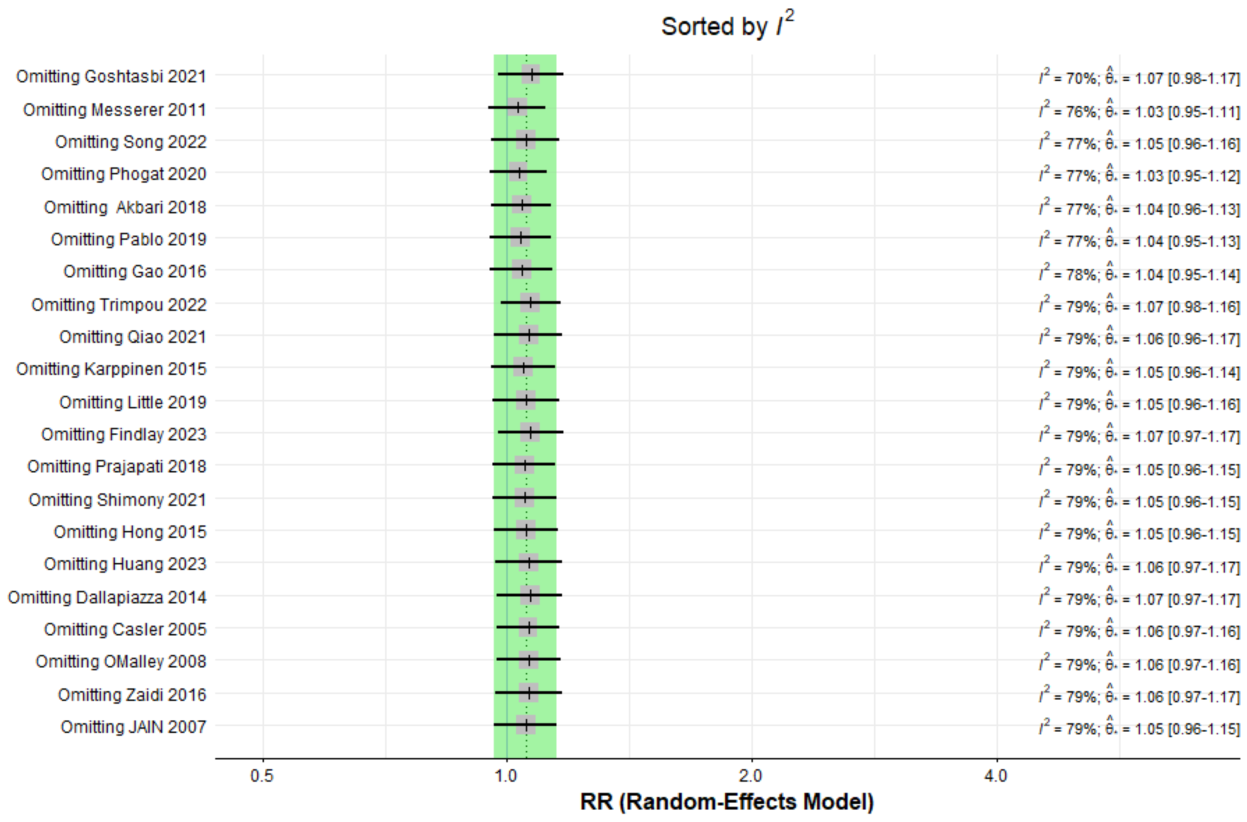

Fig 1 *Sensitivity analysis of GTR*

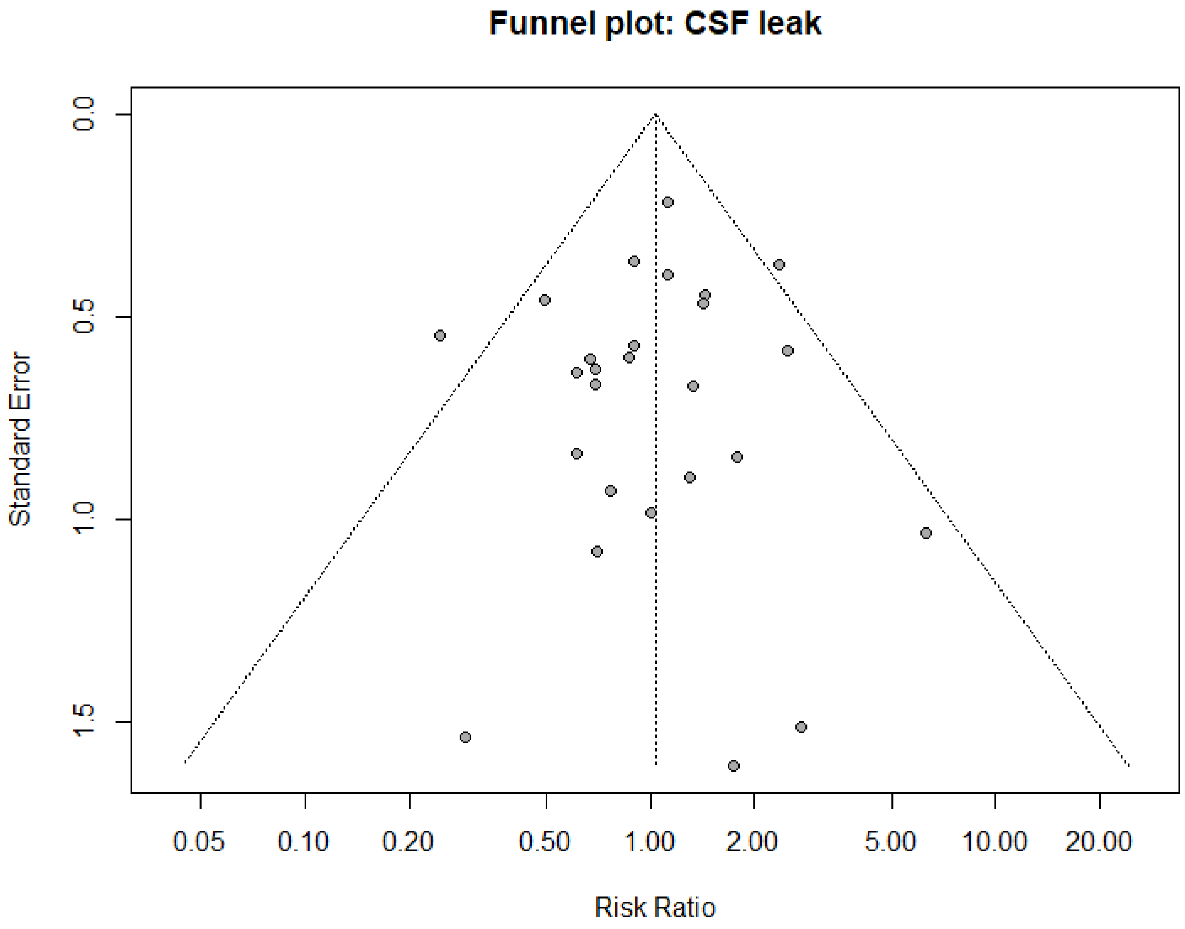

Fig 2 *Funnel plot of CSF leak*

Study	Experimental Events	Experimental Total	Control Events	Control Total	Weight	Risk Ratio MH, Random, 95% CI
Prajapati 2018	0	17	0	13	0.0%	
Zaidi 2016	0	55	0	80	0.0%	
Phogat 2020	0	119	2	79	0.6%	0.13 [0.01; 2.73]
Pablo 2019	61	140	91	259	81.6%	1.24 [0.96; 1.59]
Eseonu 2017	15	275	4	109	4.4%	1.49 [0.50; 4.38]
Findlay 2023	4	300	3	300	2.3%	1.33 [0.30; 5.91]
Agam 2018	0	170	7	983	0.6%	0.38 [0.02; 6.70]
Akbari 2018	5	16	5	19	4.7%	1.19 [0.42; 3.38]
Cheng 2011	0	68	0	59	0.0%	
Little 2019	1	177	1	82	0.7%	0.46 [0.03; 7.32]
Shimony 2021	1	39	0	48	0.5%	3.68 [0.15; 87.95]
Gompel 2021	0	261	2	273	0.6%	0.21 [0.01; 4.34]
Karppinen 2015	1	41	0	144	0.5%	10.45 [0.43; 251.67]
Messerer 2011	0	82	1	82	0.5%	0.33 [0.01; 8.06]
Halvorsen 2013	5	238	4	268	3.0%	1.41 [0.38; 5.18]
**Total (95% CI)**		**1998**		**2798**	**100.0%**	**1.22 [0.97; 1.53]**

Heterogeneity: $Tau^2 = 0$; $Chi^2 = 7.52$, df = 11 (P = 0.76); $I^2 = 0\%$ [0%; 58%]
Test for overall effect: Z = 1.73 (P = 0.08)

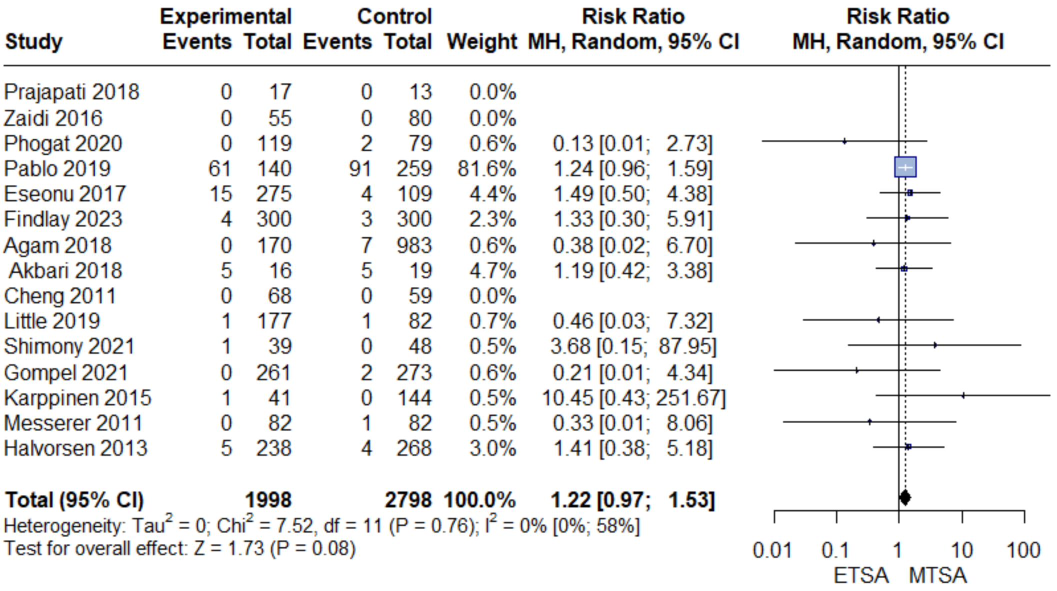

***Fig 3** Forest Plot of visual worsening*

Study	Experimental Events	Experimental Total	Control Events	Control Total	Weight	Risk Ratio MH, Random, 95% CI
Zaidi 2016	0	55	0	80	0.0%	
Trimpou 2022	0	26	0	14	0.0%	
Phogat 2020	5	119	9	79	5.6%	0.37 [0.13; 1.06]
Pablo 2019	3	140	8	259	3.7%	0.69 [0.19; 2.57]
Eseonu 2017	10	275	8	109	7.3%	0.50 [0.20; 1.22]
Findlay 2023	10	300	4	300	4.8%	2.50 [0.79; 7.88]
Agam 2018	6	170	24	983	7.7%	1.45 [0.60; 3.48]
Akbari 2018	3	16	2	19	2.4%	1.78 [0.34; 9.38]
Casler 2005	4	15	3	15	3.7%	1.33 [0.36; 4.97]
Cheng 2011	3	68	2	59	2.2%	1.30 [0.23; 7.53]
Gao 2016	6	60	5	45	5.0%	0.90 [0.29; 2.76]
Hong 2015	1	35	0	20	0.7%	1.73 [0.07; 40.59]
Huang 2023	4	73	12	54	5.4%	0.25 [0.08; 0.72]
Little 2019	6	177	4	82	4.2%	0.69 [0.20; 2.40]
Razak 2012	4	40	6	40	4.5%	0.67 [0.20; 2.18]
Shimony 2021	2	39	4	48	2.4%	0.62 [0.12; 3.18]
Gompel 2021	5	261	6	273	4.6%	0.87 [0.27; 2.82]
Karppinen 2015	1	41	5	144	1.5%	0.70 [0.08; 5.85]
Messerer 2011	10	82	7	82	7.1%	1.43 [0.57; 3.57]
Dallapiazza 2014	4	56	5	43	4.1%	0.61 [0.18; 2.15]
DHaens 2009	6	60	1	60	1.5%	6.00 [0.74; 48.34]
OMalley 2008	3	21	1	22	1.4%	3.14 [0.35; 27.88]
Kahilogullari 2013	9	25	10	25	10.9%	0.90 [0.44; 1.83]
Halvorsen 2013	12	238	12	268	9.4%	1.13 [0.52; 2.46]
**Total (95% CI)**		**2392**		**3123**	**100.0%**	**0.90 [0.69; 1.17]**

Heterogeneity: $Tau^2 = 0.0337$; $Chi^2 = 22.38$, df = 21 (P = 0.38); $I^2 = 6\%$ [0%; 39%]
Test for overall effect: Z = -0.77 (P = 0.44)

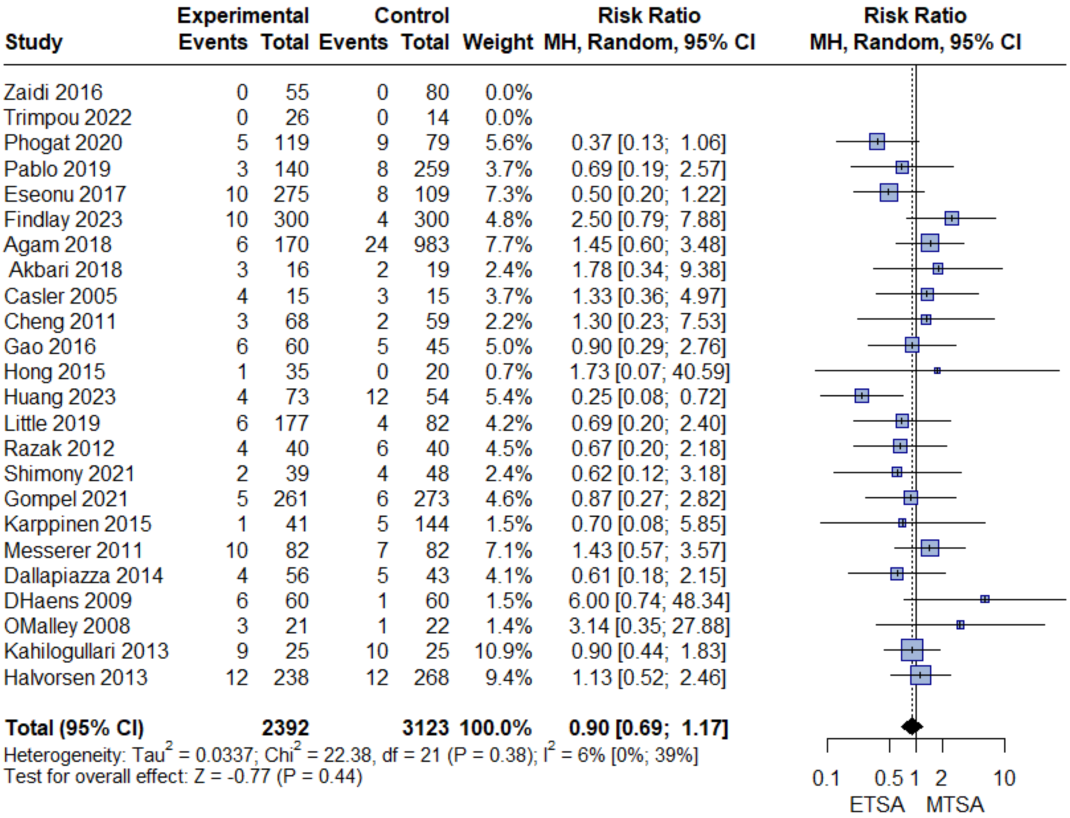

***Fig 4** Forest plot of meningitis*

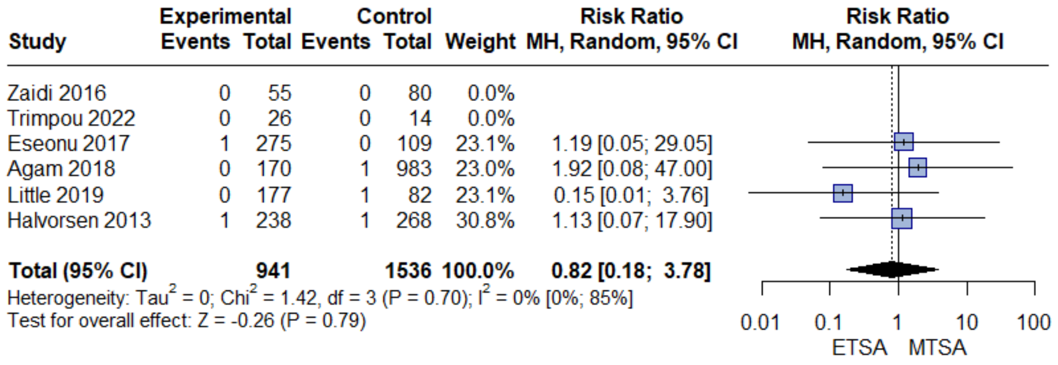

Fig 5 *Forest plot of carotid injury*

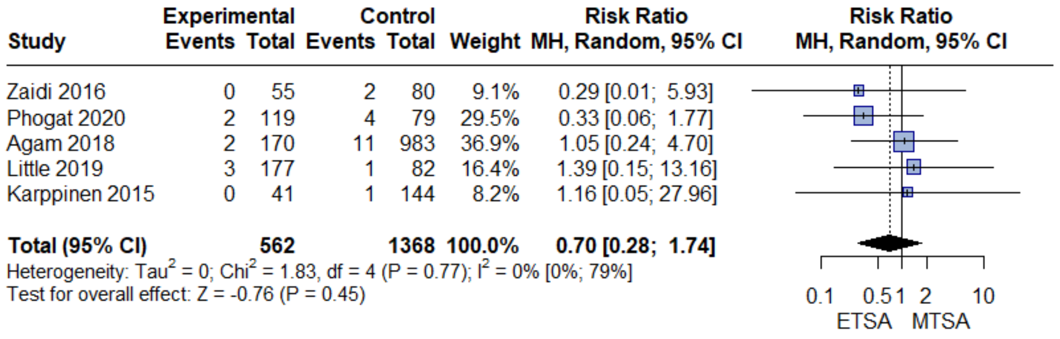

Fig 6 *Forest Plot of ICH*

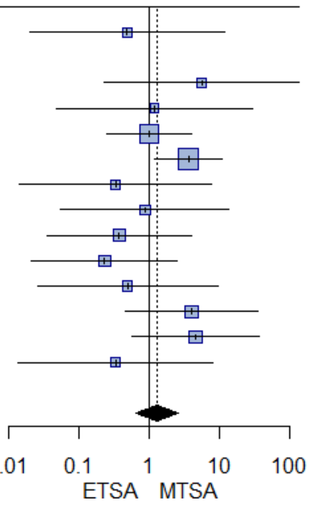

Study	Experimental Events	Experimental Total	Control Events	Control Total	Weight	Risk Ratio MH, Random, 95% CI
Zaidi 2016	0	55	1	80	4.2%	0.48 [0.02; 11.65]
Trimpou 2022	0	26	0	14	0.0%	
Pablo 2019	1	140	0	259	4.2%	5.54 [0.23; 135.12]
Eseonu 2017	1	275	0	109	4.2%	1.19 [0.05; 29.05]
Findlay 2023	4	300	4	300	16.3%	1.00 [0.25; 3.96]
Agam 2018	5	170	8	983	21.4%	3.61 [1.20; 10.92]
Casler 2005	0	15	1	15	4.3%	0.33 [0.01; 7.57]
Cheng 2011	1	68	1	59	5.4%	0.87 [0.06; 13.57]
Gao 2016	1	60	2	45	7.1%	0.38 [0.04; 4.01]
Little 2019	1	177	2	82	7.0%	0.23 [0.02; 2.52]
Karppinen 2015	0	41	3	144	4.8%	0.50 [0.03; 9.44]
Messerer 2011	4	82	1	82	8.2%	4.00 [0.46; 35.03]
Dallapiazza 2014	6	56	1	43	8.8%	4.61 [0.58; 36.85]
D'Haens 2009	0	60	1	60	4.2%	0.33 [0.01; 8.02]
**Total (95% CI)**		**1525**		**2275**	**100.0%**	**1.30 [0.66; 2.57]**

Heterogeneity: $Tau^2 = 0.2460$; $Chi^2 = 11.91$, df = 12 (P = 0.45); $I^2 = 0\%$ [0%; 57%]
Test for overall effect: Z = 0.76 (P = 0.45)

Fig 7 Forest plot of epistaxis

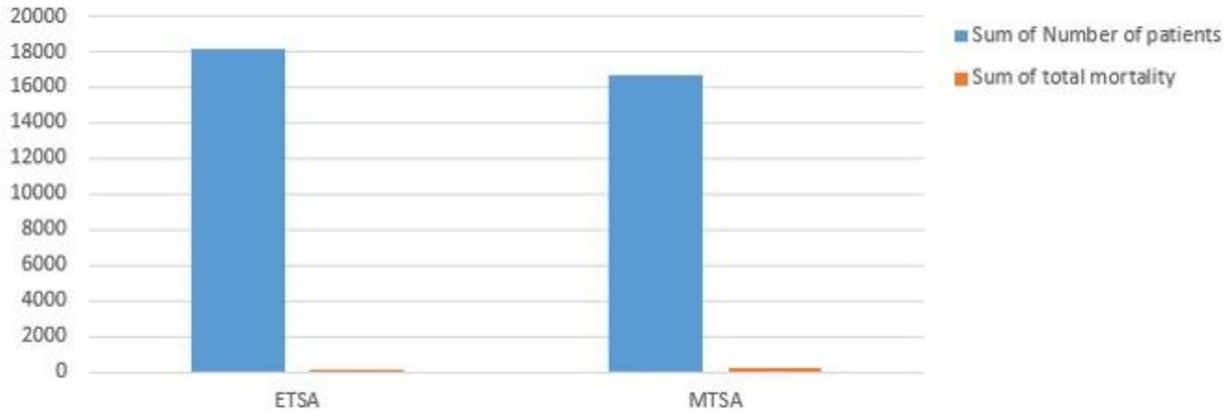

Raw Labels	Sum of Number of patients	Sum of total mortality
ETSA	18130	149
MTSA	16678	199
**Grand Total**	**34808**	**348**

Fig 8 *The bar chart of total mortality*
