# Supplementary material for: Safety and efficacy of endoscopic vs. microscopic approaches in pituitary adenoma surgery: A systematic review and meta-analysis
Source: Neurosurg Rev. 2025 Jun 1;48(1):471. doi: 10.1007/s10143-025-03600-3 (PMC12126332; doi:10.1007/s10143-025-03600-3)
Supplement: Supplementary file 2 — Supplementary file2 (PDF 262 KB) [file 10143_2025_3600_MOESM2_ESM.pdf]

Study ID	Arms	Number of patients	Age; in years; mean (SD)	Sex; Male, n (%)	Preop tumor volume in cm3; mean (SD)	Type of pituitary adenoma		Subtypes of functioning pituitary adenoma			
						functioning, n (%)	nonfunctioning, n (%)	GH-producing, n (%)	PRL-producing, n (%)	ACTH-producing, n (%)	TSH-producing, n (%)
Prajapati 2018[1]	ETSA	17	41.06 (11.75)	NR	NR	13 (43.3)	17 (56.67)	7 (23.3)	5 (16.67)	1 (3.33)	NR
	MTSA	13	41.91 (13.23)	NR	NR						NR
Zaidi 2016[2]	ETSA	55	55.9 (13.8)	35 (63.6)	13.4 (14.5)	NR	NR	NR	NR	NR	NR
	MTSA	80	59.1 (14.6)	50 (62.5)	11.0 (12.4)	NR	NR	NR	NR	NR	NR
Trimpou 2022[3]	ETSA	26	48	8 (31)	9.04 (mm)	NR	NR	NR	NR	20 (77)	NR
	MTSA	14	44	2 (14)	6.07 (mm)	NR	NR	NR	NR	5(39)	NR
Phogat 2020[4]	ETSA	119	42.3	68 (57.9)	NR	38 (23.5)	NR	NR	NR	NR	NR
	MTSA	79	44.5	49 (60.02)	NR	22 (27.8)	NR	NR	NR	NR	NR
Song 2022[5]	ETSA	210	54 (12)	100 (47.6)	2.8 (0.9)	NR	NR	NR	NR	NR	9 (4.29)
	MTSA	304	55 (12)	147 (48.4)	2.7 (0.9)	NR	NR	NR	NR	NR	17 (5.59)
Goshtasbi 2021[6]	ETSA	16373	52.8 (15.8)	8510 (52)	23.8 (12.5) (mm)	NR	NR	NR	NR	NR	NR
	MTSA	14115	53.3 (15.8)	7376 (52.3)	25.2 (13.0) (mm)	NR	NR	NR	NR	NR	NR
Pablo 2019[7]	ETSA	140	48.5 (18–85) ǂ	61 (44)	NR	NR	59 (42.14)	41 (29.28)	6 (4.29)	33 (23.6)	0
	MTSA	259	51 (17–90) ǂ	109 (42)	NR	NR	99 (38.22)	73 (28.18)	18 (6.94)	66 (25.5)	0
Eseonu 2017[8]	ETSA	275	49.0 (16.2)	115 (41.8)	6.11 (6.7)	100 (36.4)	175 (63.6)	48 (17.45)	8 (2.9)	42 (15.72)	NR
	MTSA	109	48.8 (15.8)	48 (44.0)	5.75 (6.6)	35 (32.11)	74 (67.9)	15 (13.76)	2 (1.83)	14 (12.8)	NR
Findlay 2023[9]	ETSA	300	52.8 (16.3)	123 (41)	NR	226 (75.33)	74 (24.67)	58 (19.33)	22 (7.3)	53 (17.67)	NR
	MTSA	300	52.6 (16.3)	133 (44.3)	NR	227 (75.67)	73 (24.33)	60 (20)	39 (13)	42 (14)	NR
Agam 2018[10]	ETSA	170	53.3 (13.6)	528 (45.8)	NR	NR	623 (54.03)	168 (14.6)	163 (14.14)	141 (12.22)	NR
	MTSA	983	49.1 (16.6)		NR	NR					NR
Akbari 2018[11]	ETSA	16	39.43 (15.21)	9 (56)	36.3 (4.4) (mm)	NR	NR	NR	NR	NR	NR
	MTSA	19	43.06 (11.29)	10 (53)	34.0 (4.6) (mm)	NR	NR	NR	NR	NR	NR
Casler 2005[12]	ETSA	15	41.6	6 (40)	NR	NR	NR	NR	NR	NR	NR
	MTSA	15	50.66	10 (66.67)	NR	NR	NR	NR	NR	NR	NR
Cheng 2011[13]	ETSA	68	37.2 (13 – 69) ǂ	31 (45.6)	NR	NR	NR	16 (23.5)	25 (36.77)	22 (32.35)	5 (7.3)
	MTSA	59	33.8 (11 – 71) ǂ	20 (33.9)	NR	NR	NR	13 (22.03)	25 (42.4)	18 (30.5)	3 (5.08)
Gao 2016[14]	ETSA	60	44.6 (19–75) ǂ	26 (43.33)	NR	NR	27 (45)	7 (11.67)	21 (35)	5 (8.33)	NR
	MTSA	45	48.8 (21–77) ǂ	19 (42.22)	NR	NR	22 (48.89)	4 (8.89)	16 (35.56)	3 (6.67)	NR
Higgins 2012[15]	ETSA	19	54.2	11 (58)	2.34	3 (15.79)	NR	NR	NR	NR	NR
	MTSA	29	52.8	14 (48)	2.02	11 (37.9)	NR	NR	NR	NR	NR
Hong 2015[16]	ETSA	35	50.8 (13.8)	16 (45.7)	17.3 (5.8) mm	NR	NR	NR	NR	NR	NR
	MTSA	20	58.5 (12.9)	9 (45)	22.7 (4.7) mm	NR	NR	NR	NR	NR	NR
Huang 2023[17]	ETSA	73	NR	30 (41.1)	NR	NR	15 (20.55)	8 (10.96)	7 (9.6)	12 (16.44)	NR

	MTSA	54	NR	20 (37.04)	NR	NR	11 (20.37)	5 (9.3)	8 (14.8)	7 (12.96)	NR
Little 2019[18]	ETSA	177	58.1 (14.0)	104 (58.8)	7.1 (5.7)	NR	NR	NR	9 (5.08)	NR	NR
	MTSA	82	58.6 (13.3)	52 (63.4)	7.4 (7.8)	NR	NR	NR	9 (10.98)	NR	NR
Qiao 2021[19]	ETSA	424	44.1 (12.6)	196 (46.1)	0.9 [0.4, 3.5] ǂ	NR	NR	NR	NR	NR	NR
	MTSA	694	44.2 (11.8)	318 (47.3)	0.9 [0.5, 4.0] ǂ	NR	NR	NR	NR	NR	NR
Razak 2012[20]	ETSA	40	47.4	19 (47.5)	NR	16 (40)	24 (60)	8 (20)	2 (5)	5 (12.5)	1 (2.5)
	MTSA	40	49.3	22 (55)	NR	14 (35)	26 (65)	11 (27.5)	2 (5)	1 (2.5)	0
Shimony 2021[21]	ETSA	39	57.79 (12.91)	27 (69.2)	12.1 (10.3)	NR	NR	NR	NR	NR	NR
	MTSA	48	52.75 (13.93)	30 (62.5)	9.2 (7.6)	NR	NR	NR	NR	NR	NR
Gompel 2021[22]	ETSA	261	51.7 (0.9)	NR	2.1 (0.1)	NR	116 (44.44)	38 (14.56)	17 (6.5)	58 (22.22)	4 (1.53)
	MTSA	273	50.3 (0.9)	NR	2.1 (0.1)	NR	137 (50.18)	45 (16.48)	11 (4.03)	54 (19.78)	3 (1.1)
Karppinen 2015[23]	ETSA	41	58.5 (16)	23 (56)	6.4 (4.2)	NR	NR	NR	NR	NR	NR
	MTSA	144	58.4 (13)	95 (66)	8.7 (8.2)	NR	NR	NR	NR	NR	NR
Messerer 2011[24]	ETSA	82	57 (20–82) ǂ	47 (57.32)	13190 mm3	NR	NR	NR	NR	NR	NR
	MTSA	82	56.5 (27–84) ǂ	51 (62.2)	14506 mm3	NR	NR	NR	NR	NR	NR
Dallapiazza 2014[25]	ETSA	56	56.2 (12.8)	27 (48.2)	NR	NR	NR	NR	NR	NR	NR
	MTSA	43	56.7 (16.9)	24 (55.81)	NR	NR	NR	NR	NR	NR	NR
Choe 2008[26]	ETSA	12	47 (12)	5 (41.67)	NR	NR	NR	9 (75)	NR	3 (25)	NR
	MTSA	11	48 (10)	2 (18.18)	NR	NR	NR	8 (72.7)	NR	3 (27.3)	NR
D'Haens 2009[27]	ETSA	60	37 (10-70) ǂ	19 (32)	NR	NR	NR	13 (21.67)	29 (48.33)	16 (26.67)	2 (3.33)
	MTSA	60	35 (10-68) ǂ	16 (27)	NR	NR	NR	11 (18.33)	36 (60)	13 (21.67)	0
O’Malley 2008[28]	ETSA	25	47.9 (18–73) ǂ	15 (60)	NR	NR	NR	1 (4)	3 (12)	2 (8)	1 (4)
	MTSA	25	50.8 (23–78) ǂ	16 (64)	NR	NR	NR	1 (4)	0	1 (4)	0
Jain,2007[29]	ETSA	10	40.1	NR	NR	NR	NR	NR	NR	NR	NR
	MTSA	10	31.6	NR	NR	NR	NR	NR	NR	NR	NR
Kahilogullari 2013[30]	ETSA	25	40.84 (12.56)	4 (16)	NR	NR	NR	NR	NR	NR	NR
	MTSA	25	46.56 (7.75)	6 (24)	NR	NR	NR	NR	NR	NR	NR
Halvorsen 2013[31]	ETSA	238	NR	291 (57.5)	NR	NR	352 (69.6)	79 (15.6)	20 (4.0)	51 (10.1)	4 (0.8)
	MTSA	268	NR		NR	NR					

Supplementary table 1; baseline characteristics of the included population.

ǂ; median and IQR

ETSA; Endoscopic transsphenoidal approach, MTSA; Microscopic transsphenoidal approach.

References

1. Prajapati HP, Jain SK, Sinha VD. Endoscopic versus Microscopic Pituitary Adenoma Surgery: An Institutional Experience. *Asian J Neurosurg*. 2018;13(2):217-221. doi:10.4103/ajns.AJNS_160_16

2. Zaidi HA, Awad A-W, Bohl MA, et al. Comparison of outcomes between a less experienced surgeon using a fully endoscopic technique and a very experienced surgeon using a microscopic transsphenoidal technique for pituitary adenoma. *J Neurosurg*. 2016;124(3):596-604. doi:10.3171/2015.4.JNS15102

3. Trimpou P, Backlund E, Ragnarsson O, et al. Long-Term Outcomes and Complications from Endoscopic Versus Microscopic Transsphenoidal Surgery for Cushing's Disease: A 15-Year Single-Center Study. *World Neurosurg*. 2022;166:e427-e434. doi:10.1016/j.wneu.2022.07.027

4. Phogat V, Agarwal M, Sinha VD, Purohit D. Comparative efficacy of transsphenoidal endonasal endoscopic and microscopic pituitary surgery at single center of a developing country. *J Neurol Surg B Skull Base*. 2021;82(Suppl 3):e88-e93. doi:10.1055/s-0039-3402041

5. Song S, Wang L, Qi Q, Wang H, Feng L. Endoscopic vs. microscopic transsphenoidal surgery outcomes in 514 nonfunctioning pituitary adenoma cases. *Neurosurg Rev*. 2022;45(3):2375-2383. doi:10.1007/s10143-022-01732-4

6. Goshtasbi K, Lehrich BM, Abouzari M, et al. Endoscopic versus nonendoscopic surgery for resection of pituitary adenomas: a national database study. *J Neurosurg*. 2021;134(3):816-824. doi:10.3171/2020.1.JNS193062

7. Pablo A, Sofia B, Maximiliano T, et al. Endoscopic versus Microscopic Pituitary Adenoma Surgery: A Single-center Study. *Neurol India*. 2019;67(4):1015-1021. doi:10.4103/0028-3886.266241

8. Eseonu CI, ReFaey K, Rincon-Torroella J, et al. Endoscopic versus microscopic transsphenoidal approach for pituitary adenomas: comparison of outcomes during the transition of methods of a single surgeon. *World Neurosurg*. 2017;97:317-325. doi:10.1016/j.wneu.2016.09.120

9. Findlay MC, Drexler R, Khan M, et al. A Multicenter, Propensity Score-Matched Assessment of Endoscopic Versus Microscopic Approaches in the Management of Pituitary Adenomas. *Neurosurgery*. 2023;93(4):794-801. doi:10.1227/neu.0000000000002497

10. Agam MS, Wedemeyer MA, Wrobel B, Weiss MH, Carmichael JD, Zada G. Complications associated with microscopic and endoscopic transsphenoidal pituitary surgery: experience of 1153 consecutive cases treated at a single tertiary care pituitary center. *J Neurosurg*. 2019;130(5):1576-1583. doi:10.3171/2017.12.JNS172318

11. Akbari H, Malek M, Ghorbani M, et al. Clinical outcomes of endoscopic versus microscopic trans-sphenoidal surgery for large pituitary adenoma. *Br J Neurosurg*. 2018;32(2):206-209. doi:10.1080/02688697.2018.1429569

12. Casler JD, Doolittle AM, Mair EA. Endoscopic surgery of the anterior skull base. *Laryngoscope*. 2005;115(1):16-24. doi:10.1097/01.mlg.0000150681.68355.85

13. Cheng RX, Tian HL, Gao WW, Li ZQ. A comparison between endoscopic trans-sphenoidal surgery and traditional trans-sphenoidal microsurgery for functioning pituitary adenomas. *J Int Med Res*. 2011;39(5):1985-1993. doi:10.1177/147323001103900545

14. Gao Y, Zheng H, Xu S, et al. Endoscopic versus microscopic approach in pituitary surgery. *J Craniofac Surg*. 2016;27(2):e157-9. doi:10.1097/SCS.0000000000002401

15. Higgins TS, Courtemanche C, Karakla D, et al. Analysis of transnasal endoscopic versus transseptal microscopic approach for excision of pituitary tumors. *Am J Rhinol*. 2008;22(6):649-652. doi:10.2500/ajr.2008.22.3246

16. Hong SD, Nam DH, Seol HJ, et al. Endoscopic binostril versus transnasal transseptal microscopic pituitary surgery: Sinonasal quality of life and olfactory function. *Am J Rhinol Allergy*. 2015;29(3):221-225. doi:10.2500/ajra.2015.29.4165

17. Huang Y, Zheng T, Liu Y, Fang R. Original Article Comparison of microscopic transsphenoidal surgery and neuroendoscopic transsphenoidal surgery in pituitary adenoma resection and the risk factors of postoperative cerebrospinal fluid leakage.

18. Little AS, Kelly DF, White WL, et al. Results of a prospective multicenter controlled study comparing surgical outcomes of microscopic versus fully endoscopic transsphenoidal surgery for nonfunctioning pituitary adenomas: the Transsphenoidal Extent of Resection (TRANSSPHER) Study. *J Neurosurg*. 2020;132(4):1043-1053. doi:10.3171/2018.11.JNS181238

19. Qiao N, Shen M, He W, et al. Comparative effectiveness of endoscopic versus microscopic transsphenoidal surgery for patients with growth hormone secreting pituitary adenoma: An emulated trial. *Clin Neurol Neurosurg*. 2021;207:106781. doi:10.1016/j.clineuro.2021.106781

20. Razak AA, Horridge M, Connolly DJ, et al. Comparison of endoscopic and microscopic trans-sphenoidal pituitary surgery: early results in a single centre. *Br J Neurosurg*. 2013;27(1):40-43. doi:10.3109/02688697.2012.703353

21. Shimony N, Popovits N, Shofty B, Abergel A, Ram Z, Grossman R. Endoscopic transsphenoidal surgery reduces the need for re-operation compared to the microscopic approach in pituitary macroadenomas. *Eur J Surg Oncol*. 2021;47(6):1352-1356. doi:10.1016/j.ejso.2021.02.004

22. Van Gompel JJ, Atkinson JLD, Choby G, et al. Pituitary tumor surgery: comparison of endoscopic and microscopic techniques at a single center. *Mayo Clin Proc*. 2021;96(8):2043-2057. doi:10.1016/j.mayocp.2021.03.028

23. Karppinen A, Kivipelto L, Vehkavaara S, et al. Transition from microscopic to endoscopic transsphenoidal surgery for nonfunctional pituitary adenomas. *World Neurosurg*. 2015;84(1):48-57. doi:10.1016/j.wneu.2015.02.024

24. Messerer M, De Battista JC, Raverot G, et al. Evidence of improved surgical outcome following endoscopy for nonfunctioning pituitary adenoma removal. *Neurosurg Focus*. 2011;30(4):E11. doi:10.3171/2011.1.FOCUS10308

25. Dallapiazza R, Bond AE, Grober Y, et al. Retrospective analysis of a concurrent series of microscopic versus endoscopic transsphenoidal surgeries for Knosp Grades 0-2 nonfunctioning pituitary macroadenomas at a single institution. *J Neurosurg*. 2014;121(3):511-517. doi:10.3171/2014.6.JNS131321

26. Choe J-H, Lee K-S, Jeun S-S, Cho J-H, Hong Y-K. Endocrine outcome of endoscopic endonasal transsphenoidal surgery in functioning pituitary adenomas. *J Korean Neurosurg Soc*. 2008;44(3):151-155. doi:10.3340/jkns.2008.44.3.151

27. D'Haens J, Van Rompaey K, Stadnik T, Haentjens P, Poppe K, Velkeniers B. Fully endoscopic transsphenoidal surgery for functioning pituitary adenomas: a retrospective comparison with traditional transsphenoidal microsurgery in the same institution. *Surg Neurol*. 2009;72(4):336-340. doi:10.1016/j.surneu.2009.04.012

28. O'Malley BW, Grady MS, Gabel BC, et al. Comparison of endoscopic and microscopic removal of pituitary adenomas: single-surgeon experience and the learning curve. *Neurosurg Focus*. 2008;25(6):E10. doi:10.3171/FOC.2008.25.12.E10

29. Jain AK, Gupta AK, Pathak A, Bhansali A, Bapuraj JR. Excision of pituitary adenomas: randomized comparison of surgical modalities. *Br J Neurosurg*. 2007;21(4):328-331. doi:10.1080/02688690701395447

30. Kahilogullari G, Beton S, Al-Beyati ESM, et al. Olfactory functions after transsphenoidal pituitary surgery: endoscopic versus microscopic approach. *Laryngoscope*. 2013;123(9):2112-2119. doi:10.1002/lary.24037

31. Halvorsen H, Ramm-Pettersen J, Josefsen R, et al. Surgical complications after transsphenoidal microscopic and endoscopic surgery for pituitary adenoma: a consecutive series of 506 procedures. *Acta Neurochir (Wien)*. 2014;156(3):441-449. doi:10.1007/s00701-013-1959-7
