# Supplementary material for: Safety and efficacy of endoscopic vs. microscopic approaches in pituitary adenoma surgery: A systematic review and meta-analysis
Source: Neurosurg Rev. 2025 Jun 1;48(1):471. doi: 10.1007/s10143-025-03600-3 (PMC12126332; doi:10.1007/s10143-025-03600-3)
Supplement: Supplementary file 3 — Supplementary file3 (PDF 246 KB) [file 10143_2025_3600_MOESM3_ESM.pdf]

Study ID	Cohort studies								
	Selection				Comparability	Outcome			Quality Score
	Representativeness of the exposed cohort	Selection of the non-exposed cohort	Ascertainment of exposure	Demonstration that outcome of interest was not present at the start of the study	Comparability of cohorts on the basis of the design or analysis	Assessment of outcome	Was follow-up long enough for outcomes to occur	Adequacy of follow-up of cohorts	
Agam 2018[1]	*		*		*	*	*		Fair
Akbari 2018[2]	*	*	*	*	**	*		*	Good
Casler 2005[3]	*	*	*	*	*	*		*	Good
Cheng 2011[4]	*	*	*	*	**	*	*	*	Good
Choe 2008[5]	*	*	*	*	**	*	*	*	Good
D'Haens 2009[6]	*	*	*	*	**	*	*	*	Good
Dallapiazza 2014[7]	*	*	*	*	**	*	*	*	Good
Eseonu 2017[8]		*	*	*	*	*	*		Good
Findlay 2023[9]	*	*	*	*	**	*	*	*	Good
Gao 2016[10]	*		*	*	*	*		*	Good
Gompel 2021[11]	*	*	*	*	**	*	*	*	Good
Goshtasbi 2021[12]	*	*	*	*	**	*	*	*	Good
Halvorsen 2013[13]	*	*	*	*	**	*	*	*	Good
Higgins 2012[14]		*	*	*	**	*		*	Good

Hong 2015[15]	*		*	*	*	*	*		Good
Huang 2023[16]	*	*	*	*	**	*	*	*	Good
Kahilogullari 2013[17]			*	*	*	*		*	Fair
Karppinen 2015[18]	*	*	*	*	**	*	*	*	Good
Little 2019[19]	*		*	*	**	*	*	*	Good
Messerer 2011[20]		*	*	*	**	*	*	*	Good
O'Malley 2008[21]		*	*	*	**	*	*	*	Good
Pablo 2019[22]	*	*	*	*	**	*	*	*	Good
Phogat 2020[23]	*	*	*	*	**	*	*	*	Good
Prajapati 2018[24]		*	*	*	**	*	*	*	Good
Qiao 2021[25]	*	*	*	*	**	*	*	*	Good
Razak 2012[26]			*	*	*	*			Poor
Shimony 2021[27]			*	*	*	*	*		Fair
Song 2022[28]	*	*	*	*	**	*	*	*	Good
Trimpou 2022[29]	*	*	*	*	**	*	*	*	Good
Zaidi 2016[30]	*	*	*	*	*	*	*	*	Good

Supplementary Table 2: Assessment of risk of bias using NOS

Refrences

1. Agam MS, Wedemeyer MA, Wrobel B, Weiss MH, Carmichael JD, Zada G. Complications associated with microscopic and endoscopic transsphenoidal pituitary surgery: experience of 1153 consecutive cases treated at a single tertiary care pituitary center. *J Neurosurg*. 2019;130(5):1576-1583. doi:10.3171/2017.12.JNS172318

2. Akbari H, Malek M, Ghorbani M, et al. Clinical outcomes of endoscopic versus microscopic trans-sphenoidal surgery for large pituitary adenoma. *Br J Neurosurg*. 2018;32(2):206-209. doi:10.1080/02688697.2018.1429569

3. Casler JD, Doolittle AM, Mair EA. Endoscopic surgery of the anterior skull base. *Laryngoscope*. 2005;115(1):16-24. doi:10.1097/01.mlg.0000150681.68355.85

4. Cheng RX, Tian HL, Gao WW, Li ZQ. A comparison between endoscopic trans-sphenoidal surgery and traditional trans-sphenoidal microsurgery for functioning pituitary adenomas. *J Int Med Res*. 2011;39(5):1985-1993. doi:10.1177/147323001103900545

5. Choe J-H, Lee K-S, Jeun S-S, Cho J-H, Hong Y-K. Endocrine outcome of endoscopic endonasal transsphenoidal surgery in functioning pituitary adenomas. *J Korean Neurosurg Soc*. 2008;44(3):151-155. doi:10.3340/jkns.2008.44.3.151

6. D'Haens J, Van Rompaey K, Stadnik T, Haentjens P, Poppe K, Velkeniers B. Fully endoscopic transsphenoidal surgery for functioning pituitary adenomas: a retrospective comparison with traditional transsphenoidal microsurgery in the same institution. *Surg Neurol*. 2009;72(4):336-340. doi:10.1016/j.surneu.2009.04.012

7. Dallapiazza R, Bond AE, Grober Y, et al. Retrospective analysis of a concurrent series of microscopic versus endoscopic transsphenoidal surgeries for Knosp Grades 0-2 nonfunctioning pituitary macroadenomas at a single institution. *J Neurosurg*. 2014;121(3):511-517. doi:10.3171/2014.6.JNS131321

8. Eseonu CI, ReFaey K, Rincon-Torroella J, et al. Endoscopic versus microscopic transsphenoidal approach for pituitary adenomas: comparison of outcomes during the transition of methods of a single surgeon. *World Neurosurg*. 2017;97:317-325. doi:10.1016/j.wneu.2016.09.120

9. Findlay MC, Drexler R, Khan M, et al. A Multicenter, Propensity Score-Matched Assessment of Endoscopic Versus Microscopic Approaches in the Management of Pituitary Adenomas. *Neurosurgery*. 2023;93(4):794-801. doi:10.1227/neu.0000000000002497

10. Gao Y, Zheng H, Xu S, et al. Endoscopic versus microscopic approach in pituitary surgery. *J Craniofac Surg*. 2016;27(2):e157-9. doi:10.1097/SCS.0000000000002401

11. Van Gompel JJ, Atkinson JLD, Choby G, et al. Pituitary tumor surgery: comparison of endoscopic and microscopic techniques at a single center. *Mayo Clin Proc*. 2021;96(8):2043-2057. doi:10.1016/j.mayocp.2021.03.028

12. Goshtasbi K, Lehrich BM, Abouzari M, et al. Endoscopic versus nonendoscopic surgery for resection of pituitary adenomas: a national database study. *J Neurosurg*. 2021;134(3):816-824. doi:10.3171/2020.1.JNS193062

13. Halvorsen H, Ramm-Pettersen J, Josefsen R, et al. Surgical complications after transsphenoidal microscopic and endoscopic surgery for pituitary adenoma: a consecutive series of 506 procedures. *Acta Neurochir (Wien)*. 2014;156(3):441-449. doi:10.1007/s00701-013-1959-7

14. Higgins TS, Courtemanche C, Karakla D, et al. Analysis of transnasal endoscopic versus transseptal microscopic approach for excision of pituitary tumors. *Am J Rhinol*. 2008;22(6):649-652. doi:10.2500/ajr.2008.22.3246

15. Hong SD, Nam DH, Seol HJ, et al. Endoscopic binostril versus transnasal transseptal microscopic pituitary surgery: Sinonasal quality of life and olfactory function. *Am J Rhinol Allergy*. 2015;29(3):221-225. doi:10.2500/ajra.2015.29.4165

16. Huang Y, Zheng T, Liu Y, Fang R. Original Article Comparison of microscopic transsphenoidal surgery and neuroendoscopic transsphenoidal surgery in pituitary adenoma resection and the risk factors of postoperative cerebrospinal fluid leakage.

17. Kahilogullari G, Beton S, Al-Beyati ESM, et al. Olfactory functions after transsphenoidal pituitary surgery: endoscopic versus microscopic approach. *Laryngoscope*. 2013;123(9):2112-2119. doi:10.1002/lary.24037

18. Karppinen A, Kivipelto L, Vehkavaara S, et al. Transition from microscopic to endoscopic transsphenoidal surgery for nonfunctional pituitary adenomas. *World Neurosurg*. 2015;84(1):48-57. doi:10.1016/j.wneu.2015.02.024

19. Little AS, Kelly DF, White WL, et al. Results of a prospective multicenter controlled study comparing surgical outcomes of microscopic versus fully endoscopic transsphenoidal surgery for nonfunctioning pituitary adenomas: the Transsphenoidal Extent of Resection (TRANSSPHER) Study. *J Neurosurg*. 2020;132(4):1043-1053. doi:10.3171/2018.11.JNS181238

20. Messerer M, De Battista JC, Raverot G, et al. Evidence of improved surgical outcome following endoscopy for nonfunctioning pituitary adenoma removal. *Neurosurg Focus*. 2011;30(4):E11. doi:10.3171/2011.1.FOCUS10308

21. O'Malley BW, Grady MS, Gabel BC, et al. Comparison of endoscopic and microscopic removal of pituitary adenomas: single-surgeon experience and the learning curve. *Neurosurg Focus*. 2008;25(6):E10. doi:10.3171/FOC.2008.25.12.E10

22. Pablo A, Sofia B, Maximiliano T, et al. Endoscopic versus Microscopic Pituitary Adenoma Surgery: A Single-center Study. *Neurol India*. 2019;67(4):1015-1021. doi:10.4103/0028-3886.266241

23. Phogat V, Agarwal M, Sinha VD, Purohit D. Comparative efficacy of transsphenoidal endonasal endoscopic and microscopic pituitary surgery at single center of a developing country. *J Neurol Surg B Skull Base*. 2021;82(Suppl 3):e88-e93. doi:10.1055/s-0039-3402041

24. Prajapati HP, Jain SK, Sinha VD. Endoscopic versus Microscopic Pituitary Adenoma Surgery: An Institutional Experience. *Asian J Neurosurg*. 2018;13(2):217-221. doi:10.4103/ajns.AJNS_160_16

25. Qiao N, Shen M, He W, et al. Comparative effectiveness of endoscopic versus microscopic transsphenoidal surgery for patients with growth hormone secreting pituitary adenoma: An emulated trial. *Clin Neurol Neurosurg*. 2021;207:106781. doi:10.1016/j.clineuro.2021.106781

26. Razak AA, Horridge M, Connolly DJ, et al. Comparison of endoscopic and microscopic trans-sphenoidal pituitary surgery: early results in a single centre. *Br J Neurosurg*. 2013;27(1):40-43. doi:10.3109/02688697.2012.703353

27. Shimony N, Popovits N, Shofty B, Abergel A, Ram Z, Grossman R. Endoscopic transsphenoidal surgery reduces the need for re-operation compared to the microscopic approach in pituitary macroadenomas. *Eur J Surg Oncol*. 2021;47(6):1352-1356. doi:10.1016/j.ejso.2021.02.004

28. Song S, Wang L, Qi Q, Wang H, Feng L. Endoscopic vs. microscopic transsphenoidal surgery outcomes in 514 nonfunctioning pituitary adenoma cases. *Neurosurg Rev*. 2022;45(3):2375-2383. doi:10.1007/s10143-022-01732-4

29. Trimpou P, Backlund E, Ragnarsson O, et al. Long-Term Outcomes and Complications from Endoscopic Versus Microscopic Transsphenoidal Surgery for Cushing's Disease: A 15-Year Single-Center Study. *World Neurosurg*. 2022;166:e427-e434. doi:10.1016/j.wneu.2022.07.027

30. Zaidi HA, Awad A-W, Bohl MA, et al. Comparison of outcomes between a less experienced surgeon using a fully endoscopic technique and a very experienced surgeon using a microscopic transsphenoidal technique for pituitary adenoma. *J Neurosurg*. 2016;124(3):596-604. doi:10.3171/2015.4.JNS15102
