# Supplementary material for: Safety and efficacy of endoscopic vs. microscopic approaches in pituitary adenoma surgery: A systematic review and meta-analysis
Source: Neurosurg Rev. 2025 Jun 1;48(1):471. doi: 10.1007/s10143-025-03600-3 (PMC12126332; doi:10.1007/s10143-025-03600-3)
Supplement: Supplementary file 4 — Supplementary file4 (PDF 145 KB) [file 10143_2025_3600_MOESM4_ESM.pdf]

Supplementary table 3; ROB assessment using ROB-2 tool of Cochrane.

Studie ID			Jain 2007
Randomization process	Sequence generation	Sequence generation	Y
Randomization process	Allocation concealment	Allocation concealment	NI
Randomization process	Baseline balance differences	Baseline balance differences	N
Randomization process	Final decision	Final decision	Some Concerns
Deviations from intended interventions	Participants aware	Participants aware	Y
Deviations from intended interventions	Investigators aware	Investigators aware	Y
Deviations from intended interventions	Were there deviations arose because of the experimental context	Were there deviations arose because of the experimental context	NI
Deviations from intended interventions	Deviations affected the outcome	Deviations affected the outcome	NI
Deviations from intended interventions	Balance between groups	Balance between groups	NI
Deviations from intended interventions	Appropriate analysis	Appropriate analysis	NI
Deviations from intended interventions	Potential impact of the failure to analyse participants	Potential impact of the failure to analyse participants	NI
Deviations from intended interventions	Final decision	Final decision	Some Concerns
Bias in measurement of the outcome	Method of measuring the outcome inappropriate.	Method of measuring the outcome inappropriate.	N
Bias in measurement of the outcome	Differed between intervention groups.	Differed between intervention groups.	N
Bias in measurement of the outcome	Were outcome assessors aware?	Were outcome assessors aware?	Y
Bias in measurement of the outcome	Assessment of the outcome have been influenced by knowledge.	Assessment of the outcome have been influenced by knowledge.	N
Bias in measurement of the outcome	Final decision	Final decision	Low risk
Bias due to missing outcome data	Outcome data for all participants?	Outcome data for all participants?	Y
Bias due to missing outcome data	Evidence that result is not biased	Evidence that result is not biased	Y
Bias due to missing outcome data	Messiness could depend on true value	Messiness could depend on true value	N
Bias due to missing outcome data	Messiness is likely to depend on true value	Messiness is likely to depend on true value	N
Bias due to missing outcome data	Final decision	Final decision	Low risk
Bias in selection of the reported result	Results selected from multiple measurements or analysis	Results selected from multiple measurements or analysis	NI
Bias in selection of the reported result	Trial analyzed in accordance	Trial analyzed in accordance	NI
Bias in selection of the reported result	Final decision	Final decision	Some Concerns
Other bias	Other bias	Other bias	

Final decision	Final decision	Final decision	Some Concerns
overal judgment	overal judgment	overal judgment	Some Concerns

References

1. Jain AK, Gupta AK, Pathak A, Bhansali A, Bapuraj JR. Excision of pituitary adenomas: randomized comparison of surgical modalities. *Br J Neurosurg*. 2007;21(4):328-331. doi:10.1080/02688690701395447
