# Supplementary material for: Safety and efficacy of endoscopic vs. microscopic approaches in pituitary adenoma surgery: A systematic review and meta-analysis
Source: Neurosurg Rev. 2025 Jun 1;48(1):471. doi: 10.1007/s10143-025-03600-3 (PMC12126332; doi:10.1007/s10143-025-03600-3)
Supplement: Supplementary file 5 — Supplementary file5 (PDF 85 KB) [file 10143_2025_3600_MOESM5_ESM.pdf]

Supplementary Table 4 Meta-regression for different covariates predicting outcomes.

**GTR**			
	**Beta**	**SE**	**P-value**
**Age**	-0.0042	0.009	0.65
**Sex**	0.8	0.46	0.082
**Type**	0.98	0.29	**0.006***
**Preoperative tumor volume**	0.0026	0.0089	0.77
**Knosp Grade**	1.005	0.46	**0.02***
**Postoperative hypopituitarism**			
	**Beta**	**SE**	**P-value**
**Age**	0.003	0.03	0.93
**Sex**	0.11	2.54	0.97
**Type**	-1.34	1.01	0.19
**Preoperative tumor volume**	0.14	0.05	**0.003***
**Knosp Grade**	4.26	16.05	0.8
**Diabetes insipidus**			
	**Beta**	**SE**	**P-value**
**Age**	-0.012	0.02	0.65
**Sex**	-2.08	1.9	0.28
**Type**	-1.38	1.41	0.33
**Preoperative tumor volume**	0.03	0.13	0.82
**Knosp Grade**	0.78	2.6	0.76
**Visual worsening**			
	**Beta**	**SE**	**P-value**
**Age**	0.01	0.05	0.73
**Sex**	-0.31	2.53	0.9
**Type**	0.032	1.99	0.98
**Preoperative tumor volume**	0.11	0.28	0.7

**Knosp Grade**	2.27	3.7	0.54
**Meningitis**			
	**Beta**	**SE**	**P-value**
**Age**	-0.009	0.023	0.84
**Sex**	-0.1	1.22	0.93
**Type**	-0.2	1.45	0.88
**Preoperative tumor volume**	0.026	0.16	0.87
**Knosp Grade**	0.46	2.32	0.84
**CSF leak**			
	**Beta**	**SE**	**P-value**
**Age**	0.0074	0.02	0.71
**Sex**	0.71	1.11	0.52
**Type**	0.63	1.15	0.58
**Preoperative tumor volume**	-0.012	0.13	0.93
**Knosp Grade**	-0.68	2.6	0.8
